# Inhibition of Oncogenic Kinases: An In Vitro Validated Computational Approach Identified Potential Multi-Target Anticancer Compounds

**DOI:** 10.3390/biom9040124

**Published:** 2019-03-28

**Authors:** Nazia Ikram, Muhammad Usman Mirza, Michiel Vanmeert, Matheus Froeyen, Outi M. H. Salo-Ahen, Muhammad Tahir, Aamer Qazi, Sarfraz Ahmad

**Affiliations:** 1 Institute of Molecular Biology and Biotechnology, The University of Lahore, Lahore 54000, Pakistan; nax.ikram@gmail.com; 2 Centre for Research in Molecular Medicine, The University of Lahore, Lahore 54000, Pakistan; muhammad.tahir@imbb.uol.edu.pk (M.T.); aamer.qazi@imbb.uol.edu.pk (A.Q.); 3 Department of Pharmaceutical and Pharmacological Sciences, Rega Institute for Medical Research, Medicinal Chemistry, University of Leuven, B-3000 Leuven, Belgium; michiel.vanmeert@kuleuven.be (M.V.); mathy.froeyen@kuleuven.be (M.F.); 4 Structural Bioinformatics Laboratory, Faculty of Science and Engineering, Biochemistry, Åbo Akademi University, FI-20520 Turku, Finland; osalo@abo.fi; 5 Pharmaceutical Sciences Laboratory, Faculty of Science and Engineering, Pharmacy, Åbo Akademi University, FI-20520 Turku, Finland; 6 Institute of Pharmaceutical Sciences, Riphah University, Lahore 54000, Pakistan; sarfraz.ahmad@riphah.edu.pk; 7 Department of Chemistry, Faculty of Sciences, University Malaya, Kuala Lumpur 59100, Malaysia

**Keywords:** cancer, multi-target inhibitors, receptor tyrosine kinases, serine/threonine kinases, molecular dynamics simulations, molecular docking, in vitro, cell lines

## Abstract

Tumorigenesis in humans is a multistep progression that imitates genetic changes leading to cell transformation and malignancy. Oncogenic kinases play a central role in cancer progression, rendering them putative targets for the design of anti-cancer drugs. The presented work aims to identify the potential multi-target inhibitors of oncogenic receptor tyrosine kinases (RTKs) and serine/threonine kinases (STKs). For this, chemoinformatics and structure-based virtual screening approaches were combined with an in vitro validation of lead hits on both cancerous and non-cancerous cell lines. A total of 16 different kinase structures were screened against ~739,000 prefiltered compounds using diversity selection, after which the top hits were filtered for promising pharmacokinetic properties. This led to the identification of 12 and 9 compounds against RTKs and STKs, respectively. Molecular dynamics (MD) simulations were carried out to better comprehend the stability of the predicted hit kinase-compound complexes. Two top-ranked compounds against each kinase class were tested in vitro for cytotoxicity, with compound F34 showing the most promising inhibitory activity in HeLa, HepG2, and Vero cell lines with IC_50_ values of 145.46 μM, 175.48 μM, and 130.52 μM, respectively. Additional docking of F34 against various RTKs was carried out to support potential multi-target inhibition. Together with reliable MD simulations, these results suggest the promising potential of identified multi-target STK and RTK scaffolds for further kinase-specific anti-cancer drug development toward combinatorial therapies.

## 1. Introduction

In humans, tumorigenesis is a multistep process that imitates genetic changes leading toward cell transformation and malignancy [1,2]. From a total of 518 protein kinase-coding genes constituting the human kinome, ~480 are eukaryotic protein kinases and 40 are atypical protein kinases [3,4,5]. Kinases that play a crucial role in T-cell signaling and cell activation, proliferation, and differentiation include serine/threonine kinases (STKs, e.g., Chk1, ERK1, AURK, CDKs) and receptor tyrosine kinases (RTKs, e.g., EGFR, PDGFR) [6,7,8,9,10,11]. Mutations in kinases have been observed in a variety of human tumors, and their deregulation has been shown to be the cause of numerous human malignancies [12,13,14,15,16]. Signal transduction by these kinases activates transcription factors (e.g., AP1, NFκB, Myc), which in turn leads to cell proliferation or the inhibition of programmed cell death by either participating in the deregulation of the cell cycle control or by inhibiting the pro-apoptotic molecules (e.g., Bad, Bax) [6,17,18,19]. Therefore, kinases play a central role in oncogenesis and are considered potential targets for anti-cancer drug design [20,21,22,23,24,25,26].

The search for active compounds using various structure-based computational methods [27,28] has become extremely popular in the pharmaceutical industry [29]. The current trend is to improve, utilize, and combine methods that already exist, rather than to invent completely new approaches [30,31,32,33,34,35,36,37,38,39,40,41,42]. Evidence from the literature demonstrates that the in silico identification of active protein kinase inhibitors has been made possible through screening either diverse or target-focused compound libraries [43,44]. For example, an epidermal growth factor receptor (EGFR) inhibitor was discovered through in silico docking with the crystal structure of the receptor [45]. Novel cyclin-dependent kinase 2 (CDK2) inhibitors were found using a combined ligand-based and receptor-based virtual screening [46]. Potential inhibitors against Chk1 were identified by docking a large library of compounds into the ATP binding site of the Chk1 crystal structure after excluding compounds with six or more rotatable bonds and a molecular weight of more than 550 Da [47]. Another study reported how a novel CDK inhibitor scaffold was discovered using high-throughput docking and subsequently optimized into selective binders for a CDK2 isoform based on available information from crystallographic complexes and CDK4 inhibitor structures [48]. Inhibitors for CDK2, polo-like kinase 1 (PLK1), and casein kinase 2 (CK2) have also been discovered through high-throughput docking [49,50]. Moreover, virtual screening using pharmacophore models identified selective glycogen synthase kinase-3 (GSK-3) inhibitors [51]. A dual inhibitor of Src and vascular endothelial growth factor receptor 2 (VEGFR2) that interacts with the conserved C-helix glutamate was discovered by rational structure-based design strategies, and is now in clinical trials (TG-100801) [52]. Targeting the mitogen-activated protein kinase (MEK) allosteric inhibitor site through comparative molecular field analysis (CoMFA) has helped in prioritizing synthesis efforts, [53] while the optimization of biphenyl amides as allosteric inhibitors of p38 has been guided by visual examination of the crystallographic binding modes [54]. By employing computational approaches such as e-pharmacophore modeling and docking-based virtual screening, several novel kinase inhibitors have been identified against Aurora-A kinase, p38α mitogen-activated protein kinases (p38α MAPKs), MEK1, c-Jun N-terminal kinase 1 (JNK1 or MAPK8), Mast/stem cell growth factor receptor (SCFR or c-KIT), and protein kinase B (PKB) [55,56,57,58,59,60,61]. Similarly, the 3D-QSAR (three-dimensional quantitative structure–activity relationship) technique has been used for several successful in silico screening efforts [62,63,64,65,66].

In drug development (especially in the case of kinase inhibitors), several ‘complexity’ issues have resulted in reduced efficacies, resistance profiles, and undesired safety concerns of multiple drugs that are typically being developed for one single target. Such issues include signaling network robustness, ref. [67] crosstalk between different signaling pathways, ref. [68] redundancy in transcription networks, ref. [69] compensatory and neutralizing actions in signaling pathways [70], on-target and off-target toxicities of the candidate inhibitors [71], and anti-target and counter-target activities induced by drugs [72]. In order to improve the safety and resistance profiles, together with enhancing therapeutic efficacies, multi-target agents and drug combinations are highly sought after [67,73,74]. Successful clinical anti-cancer multi-target kinase inhibitors include: sunitinib against platelet-derived growth factor receptor (PDGFR) and VEGFR, dasatinib against Abl and Src, sorafenib against Braf and VEGFR, and lapatinib against epidermal growth factor receptor (EGFR) and human epidermal growth factor receptor 2 (HER2 or ERBB2). Whilst multi-kinase inhibitors are actively being pursued by current drug discovery efforts [75,76], novel search methods for efficient multi-target agents are highly desired [77,78]. Virtual screening methods have been mostly explored against individual targets [30,42,79] using a combination of molecular docking and pharmacophore and QSAR modeling [80,81] Only a limited number of multi-target virtual screening approaches have been reported and critically reviewed by Knight et al. [20], Dar et al. [82], Jalencas and Mestres [83], and recently by Zhang et al. [84].

We focus on important kinase targets, RTKs and STKs, involved in a broad range of cancers. Here, we present a systematic, hierarchical structure-based virtual screening (SBVS) workflow to identify kinase inhibitors as anti-cancer compounds with potential multi-target properties. The dynamic properties of the top-ranked ligand-target complexes are then studied by molecular dynamics (MD) simulations to assess their stability and elucidate the significant binding interactions. Finally, experimental validation of the virtual hits is carried out in vitro in common cancerous and non-cancerous cell lines. This methodological study resulted in promising molecules that may serve as leads for drugs that can help combat a wide range of cancers.

## 2. Material and Methods

### 2.1. Computational Methods

#### 2.1.1. Protein Dataset Preparation

Protein kinases have an ATP binding site that is located in the hinge region between the N-terminal and C-terminal lobes [5]. A wide range of crystal structures of different kinases complexed with reported kinase inhibitors is present in the Protein Data Bank (PDB) [85]. For the current study, crystal structures of RTKs (Supplementary Table S1) and STKs (Supplementary Table S2) were retrieved from the PDB. All the protein structures were prepared for structure-based virtual screening (SBVS). Hydrogen atoms were added and co-crystallized water molecules and small molecules were removed, followed by energy minimization and optimization using the DockPrep module of UCSF Chimera 10.1 (Resource for Biocomputing, Visualization, and Informatics, University of California San Francisco, CA, USA) [86]. Details of protein preparation, optimization, and minimization before the ensemble docking are described elsewhere [35,37,87]. Briefly, all the heteroatoms and solvent molecules were removed, while Gasteiger charges and hydrogens were added using Chimera. The structure was minimized for 1000 steepest descent steps with a root mean square gradient of 0.02 having an update interval of 10 and using the AMBER ff14SB force field.

#### 2.1.2. Ligand Preparation

A chemical library of ~15.4 million molecules was retrieved from PubChem BioAssay and ZINC15 databases for the SBVS study [88,89]. Prior to this, drug-like filters were applied to generally assess the oral bioavailability, solubility, and safety of the compounds. These include Lipinski’s Ro5, ref. [90] Pfizer 3/75 (logP >3 and topological polar surface area, TPSA <75 Å^2^) [91], GSK 4/400 [92] (‘bad’ or ‘good’ ADMET profile [absorption, distribution, metabolism, elimination, toxicity] including: logP ≤4 and molecular weight ≤400) and Veber Rule [93] (‘bad’ or ‘good’ oral bioavailability rule including: rotatable bonds ≤10 and TPSA ≤140 Å^2^ or H-bond donors and H-bond acceptors ≤12). From these subsets, the duplicate structures were removed to acquire unique scaffolds based on the InChlKey identifiers generated by Open Babel [94]. Specific filters were set to screen for optimal pharmacokinetic parameters for good human intestinal absorption (HIA) [95] and blood–brain barrier (BBB) permeability [96]. Further, a series of PAINS filters (pan assay interference compounds) filters (I, II, and III) [97] were applied to remove the compounds with functional groups that can interfere with many screening assays. Additionally, toxicophore-containing molecules with reactive functional groups were filtered out using the Brenk structural alert [98] and REOS (Rapid Elimination Of Swill) filters [99,100]. The resulted dataset of compounds was finally used for SBVS. To better comprehend the contribution of interacting residues lining the groove of the active site, a binding site analysis was carried out by comparing several crystallized small compounds inside the kinase ATP-binding site with high affinity and specificity, as reported by numerous studies [101,102,103,104].

#### 2.1.3. Virtual Screening

After the protein and ligand dataset preparation, docking-site grids were created with the grid generation panel of AutoDock Vina [105] and relaxed on the investigated binding site (active site) of the prepared proteins. Autodock Vina was declared among the top-ranked scoring functions in terms of docking power and screening test according to CASF (Comparative Assessment of Scoring Functions) benchmark 2013 [106]. Accordingly, a grid was made for each protein with cubic dimensions of 30 Å × 30 Å × 30 Å, covering the whole N-terminal region of the intracellular kinase domain. Prior to the actual ensemble docking studies, a cognate study was set up to validate the accuracy of the docking protocol used in the SBVS workflow. The representative crystal structures for each kinase protein complexed with an inhibitor were selected according to the following criteria: (i) resolution (highest possible) and R-free factor (lowest possible); and (ii) accuracy of the binding mode prediction when re-docking the co-crystallized compound in the active site. After each co-crystalized compound was re-docked with the respective target, the root mean square deviation (RMSD) of the binding conformation with the highest binding affinity was calculated from the co-crystallized reference structure. The docking protocol was considered to generate accurate binding poses for a particular inhibitor if the observed RMSD <1.9 Å.

The filtered database (described in ligand preparation) was finally uploaded in the Mcule drug discovery platform [107] (in SMILES format). Autodock Vina, automated by the Mcule drug discovery pipeline, was used to screen the composed compound library. To reduce the size of the library and maximize the coverage of chemical space at the same time, diversity selection, an optimized implementation of the stepwise elimination algorithm (in the Mcule virtual screening pipeline), was incorporated in the workflow before docking. This procedure eliminated the closest analogs (based on the Tanimoto similarity coefficient) and maximized the diversity of the possible identified active scaffolds. The Mcule docking engine (in-built with Autodock Vina) uses a gradient optimization method in its local optimization procedure to rank the best poses efficiently. Individual ligands of the compound library were docked iteratively into the respective active sites of the representative structures of RTKs and STKs, and were ranked based on the binding affinity. The scoring of the generated docking poses and ranking of the ligands is based on the Vina empirical scoring function that approximates the ligand binding affinity in kcal/mol. Then, the top hits were subjected to an extensive in silico analysis of their pharmacokinetic properties by the QSAR-based admetSAR [108] and PreADMET servers [109]. The BBB permeation [96], HIA [95], aqueous solubility [110], Caco-2 cell permeability, cytochrome P450 (CYP450) inhibition [111], and Ames mutagenicity [112] were calculated for the compounds. The best hits with the most favorable properties complexed with representative targets were manually curated using UCSF Chimera 10.1 and LigPlot+ [87,113].

#### 2.1.4. Molecular Dynamics Simulations

Based on the molecular interactions observed in the visual inspection, the overall stability of the best complexes was examined over a period of 20 ns MD simulations using the AMBER 16.0 software package [114]. All of the MD simulations were conducted on a Linux-based (CentOS 8.0) workstation equipped with an NVIDIA GeForce GTX 1080 graphics card. Amber ff14SB force field [115] parameters were used to describe the proteins, counter ions, and water. This force field has an improved protein secondary structure balance and dynamics including side chain dihedral corrections compared to the earlier AMBER force fields such as ff99. The Antechamber package of AmberTools was employed to generate the general AMBER force field (GAFF) [116] parameters for the studied ligands (using AM1–BCC charge definitions). The tleap module was used to add hydrogens to the proteins and prepare the input topology and coordinates of the simulation system. The charges of the simulation system were neutralized by adding Na^+^ counter ions around the ligand–kinase complex that was centered in a dodecahedral TIP3P [117] water box with a 10-Å distance from the edge of the box. The particle mesh Ewald method [118] with a 10-Å cut-off for non-bonded interactions was used for computing long-range interactions in the periodic simulation system. The integration time step was set to 2 fs, and the SHAKE algorithm [119] was used to constrain hydrogen-containing bonds. Prior to the actual simulation, the energy of the system was minimized using the steepest descent and conjugate gradient methods. Then, the system was gradually heated (in 50 ps) from 0 to 300 K with positional restraints on the ligand–protein complex. After heating, the system was equilibrated for 500 ps under isothermal-isovolumetric (NVT) conditions without positional restraints, and finally, a 20 ns MD production run without positional restraints was performed using constant pressure (*p* = 1.0 atm) and temperature (T = 300 K). Coordinate trajectories were collected every 2 ps during the simulation and the CPPTRAJ program of AmberTools was employed for the trajectory analyses [120]. The RMSD between the Cα atoms of the protein and all the atoms of the ligand along the trajectory was computed to assess the complex stability. The final coordinates of the complexes after the 20 ns simulation were visually investigated to provide insights into intermolecular contacts as well as the domain/region flexibility induced upon binding of the ligand. Intermolecular contacts (hydrogen bonds and non-bonded contacts) were analyzed with the LIGPLOT software. The default settings were used: 3.9 Å distance cut-off for non-bonded contacts of heavy atoms; 2.7 Å and 3.5 Å distance cut-offs for proton (H)—acceptor (A) and donor (D)—acceptor distances, respectively, with minimum 90° angles D–H–A, H–A–AA, D–A–AA (AA = acceptor antecedent) for hydrogen bonds. Based on the extensive post-MD analysis, the best compounds were purchased, and in vitro corroborated on various cell lines.

#### 2.1.5. Comparative Docking

Since the scoring functions in all of the current docking programs are only estimating the real binding affinity, they may not perform accurately in ranking the best compounds [121,122]. Therefore, it may be useful to test several different docking programs and/or scoring functions case by case. We performed a restricted comparative docking study by docking the top hits and respective co-crystallized ligands to respective target proteins with Glide [123,124] as implemented in Schrödinger’s Maestro modeling package v. 10.7.014. Ligand and protein preparation in Maestro were carried out with LigPrep (protonation states were assigned at pH 7.0 ± 2.0 with Epik, all possible stereoisomers were created) and Protein Preparation Wizard (adding all the hydrogen atoms and missing side chains, interactive optimization of H-bonding networks, short minimization of only the added hydrogen atoms using the OPLS 3 force field), respectively. The binding-site grid box was generated with dimensions 20 Å × 20 Å × 20 Å covering the active site around the co-crystallized ligand present in the representative kinase structures. The diameter midpoint of each ligand was required to remain in a smaller box of 10 Å × 10 Å × 10 Å. The docking was carried out with the ‘extra precision’ docking mode (Glide XP). The empirical scoring function of Glide XP has been optimized by the inclusion of water desolvation terms and specific molecular recognition motifs [125,126]. The best-docked poses were further evaluated with the MM-GBSA (molecular mechanics–generalized Born surface area) tool of Prime v. 3.0 as implemented in Maestro, using OPLS 3 (Optimized Potentials for Liquid Simulations) force field and the VSGB2.1 solvation model [127]. This method provides a more accurate way of calculating the free energy of binding for receptor–ligand complexes [128,129]. Extensive post-docking analysis was performed to compare the Glide binding poses with the MD simulated AutoDock Vina poses to gain more understanding of the predicted molecular interactions.

### 2.2. MM-GBSA Calculations Using AMBER

The binding free energies (ΔG_tol_) of RTKs and STKs complexed with screened compounds were calculated using the MM-GBSA method, implemented in the AMBER 16 simulation package. For each system, 1000 snapshots were generated from the last 20 ns stable MD simulation with an interval of 2 ps. The binding free energy is calculated as a sum of the molecular mechanics binding energy (ΔE_MM_) and solvation free energy (ΔG_sol_) as follows:

(1) $$\Delta E_{\mathrm{gas}}=\Delta E_{\mathrm{int}}+\Delta E_{\mathrm{ele}}+\Delta E_{\mathrm{vdw}}$$

(2) $$\Delta G_{\mathrm{sol}}=\Delta G_{p}+\Delta G_{\mathrm{np}}$$

(3) $$\Delta G_{\mathrm{tol}}=\Delta E_{\mathrm{MM}}+\Delta G_{\mathrm{sol}}$$

(ΔE_MM_) is further divided into internal energy (ΔE_int_), electrostatic energy (ΔE_ele_), and van der Waals energy (ΔE_vdw_), whereas the total solvation free energy (ΔG_sol_) is contributed by the sum of polar (ΔG_p_) and non-polar (ΔG_np_) components.

### 2.3. Experimental Procedures: In Vitro Cytotoxicity

#### 2.3.1. Materials

HeLa (human epithelial cervical cancer, ATCC CCL-2), HepG2 (human epithelial liver cancer, ATCC HB-8065) and Vero (monkey epithelial kidney cancer, ATCC CCL-81) cell lines were used to examine the selected virtual hit compounds’ anti-cancer activity. All cell lines were generously provided by the late Prof. Dr. M.H. Qazi, University of Lahore, Lahore, Pakistan. The test compounds were purchased via MolPort and cisplatin from Pharmedic (Pvt) Ltd., Lahore, Pakistan. Cell culture media, fetal calf serum (FCS), MTT (3-[4-C-dimethylthiazol-2-yl]-2,5-diphenyltetrazolium bromide), DMSO, antibiotic/antimycotic and Supplementary Materials were purchased from Thermo Fisher Scientific, Inc, (Waltham, MA, USA).

#### 2.3.2. MTT Assay

Cells were harvested at 80% confluence by treating the medium-free monolayer with trypsin-EDTA. Cell counts were performed with a hemocytometer, and cell viability was determined with trypan blue. The cell suspension was adjusted to a final concentration of 7 × 10^5^ cells/mL in DMEM (Dulbecco’s modified Eagle medium) with 10% FCS. Cells were dispensed to tissue culture grade 96-well plates, with each microwell receiving 100 µL, i.e., 7000 cells per microwell. The plate was incubated at 37 °C for 12 h in 5% CO_2_ incubator. Stock solutions (10 mM) of the test compounds and positive control were prepared in DMSO. Test concentrations were prepared by diluting the stocks with the growth media. Test compound solutions were diluted from 100 µL to a final well volume of 200 µL. MTT reagent was prepared in phosphate-buffered saline (PBS) in a concentration of 5 mg/mL. After 48 h, 25 µL of MTT reagent was added to each microwell, followed by incubation for 5 h. MTT is reduced by metabolically active cells to insoluble purple formazan crystals by mitochondrial enzymes. The reduction of MTT is primarily due to glycolytic activity within the cell, and is dependent upon the presence of NADH and NADPH. The entire contents of each microwell were carefully pipetted out without disturbing the cells on the well floor. In order to solubilize the formazan crystals, 100 µL of DMSO was dispensed to each well. The absorbance was measured with the help of a Bio Rad PR 4100 microplate reader at a wavelength of 570 nm. Serial dilutions of cisplatin were used as a positive control, while the negative controls contained only the reagent, but not any drug. Percentage viability was calculated and dose–response curves were plotted using the percentage viability against the drug/test compound concentration (µM). Results represent mean ±SD of six readings: three determinations of two independent experiments.

## 3. Results and Discussion

### 3.1. Structure-Based Virtual Screening

**Figure 1:**
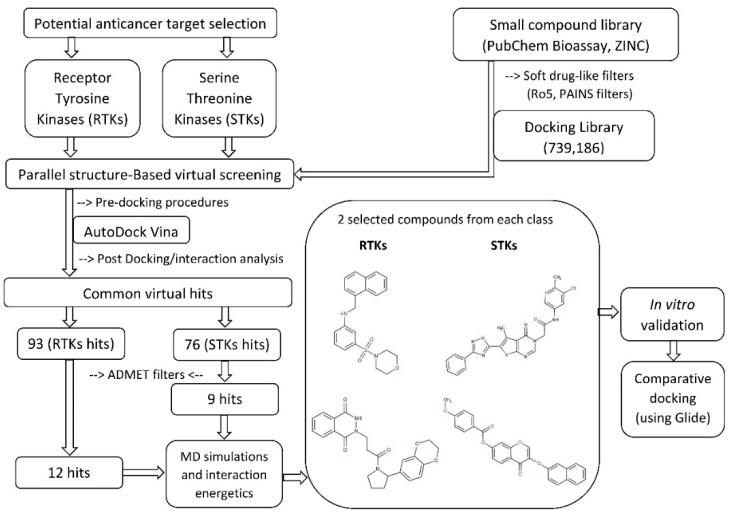
Schematic workflow is summarizing the virtual screening of kinase inhibitors.

Structure-Based Virtual Screening of large compound libraries has been successfully employed in various studies to target specific structures [60,61,85,130,131,132,133,134]. The schematic SBVS flowchart of the presented work is shown in Figure 1. Initially, a chemical library containing ~15.4 million small compounds retrieved from PubChem Bioassay and ZINC was screened, after deletion of duplicates, for drug-like properties and oral bioavailability. Additionally, a series of substructure filters were applied to remove promiscuous (PAINS) structures and compounds with toxicophoric moieties (e.g., epoxides and quinones) to ensure a favorable drug safety profile. By these criteria, a significant proportion of the initial library was excluded from the final screening library. A total of 739,186 library compounds were filtered using the diversity selection algorithm as implemented in the Mcule pipeline (Tanimoto coefficient, Tc 0.7) to maximize the diversity of the possible active scaffolds to be identified from the library, and later docked into the active-site binding pocket of each kinase. The calculated binding affinities of the docked complexes were ranked and compared with the calculated binding affinity of the re-docked co-crystallized ligand. In addition, the best-ranked binding conformations were analyzed by superposing them with the co-crystallized ligand. The binding affinity cut-off of the hit compounds was chosen to be the calculated affinity value of the re-docked co-crystallized ligand. A total of 93 and 76 virtual hits displayed a higher predicted affinity than the cut-off value against RTKs and STKs, respectively. All of the virtual hit compounds had good predicted affinities against two or more kinases, which is consistent with the structurally and evolutionary conserved binding site residues interacting in both STKs and RTKs (Supplementary Figures S1 and S2). These putative multi-target kinase inhibitors were further analyzed for their predicted pharmacokinetic properties (i.e., ADMET properties: absorption, distribution, metabolism, elimination, toxicity). Respectively 12 and 9 virtual hits against RTKs and STKs passed this series of ADMET filters. Based on the admetSAR and PreADMET predictions, all of these compounds showed favorable intestinal absorption and good BBB permeability. Moreover, these compounds were not predicted to inhibit P-glycoprotein or the most important drug-metabolizing CYP450 enzymes (1A2, 2C9, 2C19, 2D6, and 3A4). Neither did any of the compounds show an inhibitory effect on renal organic cation transporter. Furthermore, no compound was predicted to be toxic or carcinogenic based on the Ames mutagenicity test data.

### 3.2. Molecular Interactions with RTKs and STKs

**Figure 2:**
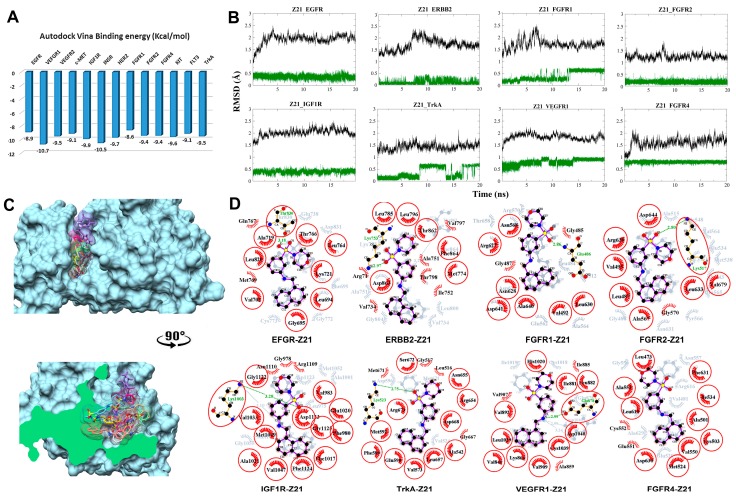
Post-molecular dynamics (MD) analysis of Z217168138–receptor tyrosine kinases (Z21–RTK) complexes. Only the most stable complexes are presented in panels (**B**–**D**). (**A**) Predicted binding affinities of Z21 docked against individual RTKs; (**B**) Root mean square deviation (RMSD) from the initial coordinates of the RTK protein’s Cα atoms (black) and all atoms of the bound Z21 (green) over a simulation time of 20 ns; (**C**) Molecular surface representation of the eight most stable complexes superimposed. The lower panel displays the cross-section of the complexes. (**D**) Two-dimensional (2D) interaction plots between Z21 and the individual RTKs, before (grey) and after MD simulation (red/purple). Residues involved in hydrophobic contacts have black labels with a spoked red arc, whilst those involved in hydrogen bonding have green labels with an H-bond distance in Ångströms. Conserved interacting residues after MD simulations are marked with red circles. Atom colors are displayed as black for carbon, red for oxygen, yellow for sulfur, and blue for nitrogen.

**Figure 3:**
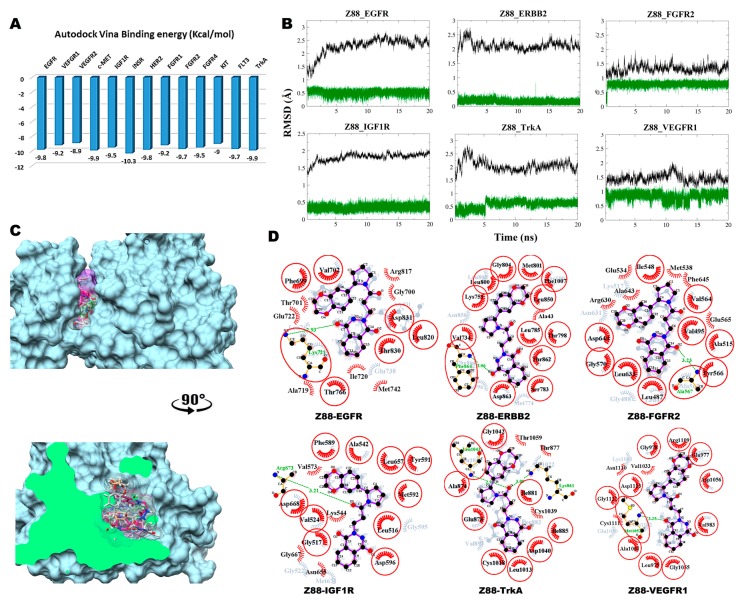
Post-MD analysis of Z88445222–receptor tyrosine kinases (Z88–RTK) complexes. Only the most stable complexes are presented in panels (**B**–**D**). (**A**) Predicted binding affinities of Z88 docked against individual RTKs; (**B**) RMSD from the initial coordinates of the RTK protein’s Cα atoms (black), and all atoms of the bound Z88 (green) over a simulation time of 20 ns; (**C**) Molecular surface representation of the six most stable complexes superimposed. Lower panel displays the cross-section of the complexes. (**D**) 2D interaction plots between Z88 and the individual RTKs, before (grey) and after MD simulation (red/purple). Residues involved in hydrophobic contacts have black labels with a spoked red arc, whilst those involved in hydrogen bonding have green labels with an H-bond distance in Ångströms. Conserved interacting residues after MD simulations are marked with red circles. Atom colors are displayed as black for carbon, red for oxygen, yellow for sulfur, and blue for nitrogen.

**Figure 4:**
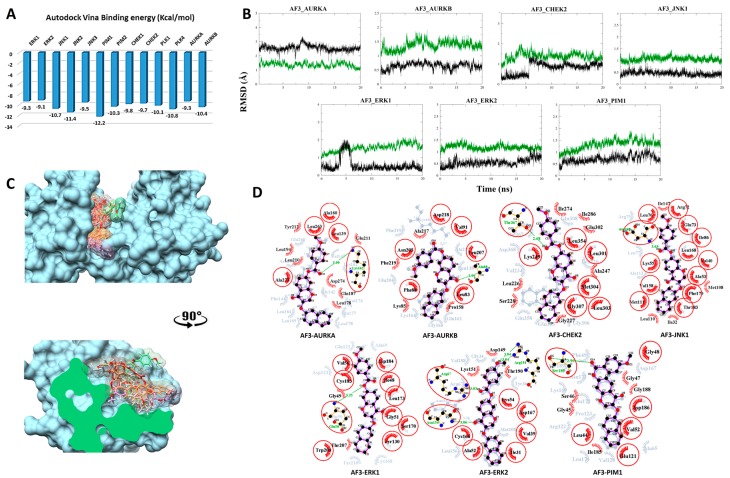
Post-MD analysis of AF-399/40714045–serine/threonine kinases (AF3–STK) complexes. Only the most stable complexes are presented in panels (**B**–**D**). (**A**) Predicted binding affinities of AF3 docked against individual STKs; (**B**) RMSD from the initial coordinates of the STK protein’s Cα atoms (black), and all atoms of the bound AF3 (green) over a simulation time of 20 ns; (**C**) Molecular surface representation of the seven most stable complexes superimposed. The lower panel displays the cross-section of the complexes. (**D**) 2D interaction plots between AF3 and the individual STKs, before (grey) and after MD simulation (red/purple). Residues involved in hydrophobic contacts have black labels with a spoked red arc, whilst those involved in hydrogen bonding have green labels with an H-bond distance in Ångströms. Conserved interacting residues after MD simulations are marked with red circles. Atom colors are displayed as black for carbon, red for oxygen, yellow for sulfur, and blue for nitrogen.

**Figure 5:**
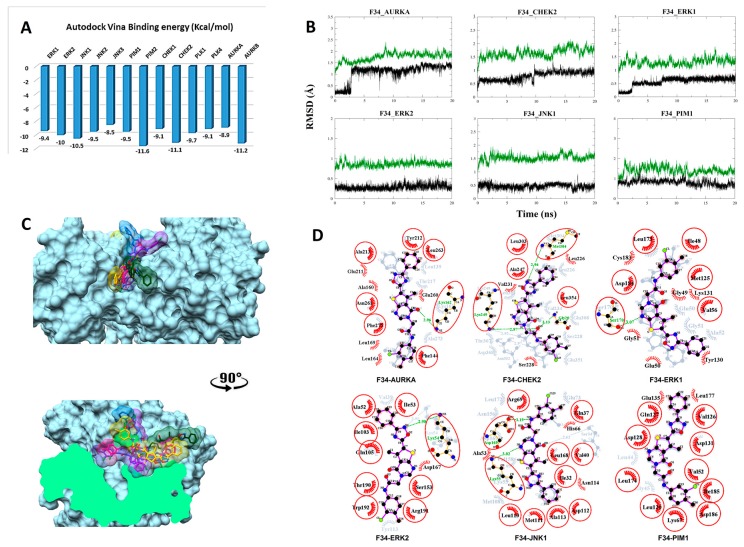
Post-MD analysis of F3411-7101–serine/threonine kinases (F34–STK) complexes. Only the most stable complexes are presented in panels (**B**–**D**). (**A**) Predicted binding affinities of F34 docked against individual STKs; (**B**) RMSD from the initial coordinates of the STK protein’s Cα atoms (black) and all atoms of the bound F34 (green) over a simulation time of 20 ns; (**C**) Molecular surface representation of the six most stable complexes superimposed. The lower panel displays the cross-section of the complexes. (**D**) 2D interaction plots between F34 and the individual STKs, before (grey) and after MD simulation (red/purple). Residues involved in hydrophobic contacts have black labels with a spoked red arc, whilst those involved in hydrogen bonding have green labels with an H-bond distance in Ångströms. Conserved interacting residues after MD simulations are marked with red circles. Atom colors are displayed as black for carbon, red for oxygen, yellow for sulfur, and blue for nitrogen.

The molecular interactions of the tested compounds with their putative target proteins (RTKs: Z21 and Z88; STKs: F34 and AF3) were carefully analyzed. The stability of the top hits docked RTK and STK complexes was studied by 20 ns MD simulations. Predicted complexes with RMSD plots that converged (RMSD spread <0.5 Å) during the last 5 ns of the 20 ns simulation were considered to be stable. Among the best compounds after post-MD extensive analysis, hit compounds available in the MolPort stock were acquired from ENAMINE Ltd., Life Chemicals Inc and Specs with catalog numbers Z217168138 (Z21) and Z88445222 (Z88) against RTKs, and F3411-7101 (F34) and AF-399/40714045 (AF3) against STKs, respectively. The detailed physicochemical and ADMET profile of all four compounds are tabulated in Supplementary Table S3. Before compound testing, an extensive interaction analysis confirmed the conservation of hydrophobic interactions and hydrogen bonds at the kinase active site as shown in Figure 2, Figure 3, Figure 4 and Figure 5.

#### 3.2.1. Compound Z21 with RTKs

The predicted binding energy after docking Z21 into RTKs indicated the strongest interaction with VEGFR1 (−10.7 kcal/mol) (Figure 2A). RMSD plots from the MD simulations for the eight most stable complexes are shown in Figure 2B. Plots for EGFR, insulin-like growth factor 1 receptor (IGF1R), ERBB2 (HER2), fibroblast growth factor receptor 1 (FGFR1), FGFR4, and VEGFR1, tropomyosin receptor kinase A (TrkA), and FGFR2 display a clear RMSD convergence during the final 5 ns, indicating complex stability. Superimposition of the eight most stable complexes after MD is displayed in Figure 2C,D. Interaction plots of the individual complexes before and after MD are shown in Figure 2D.

In EGFR, Thr830 interacts through hydrogen bond formation with the sulfonamide group of Z21. Two new Van der Waals (VDW) contacts are seen with Gln767 and Met769. In ERBB2, a VDW interaction is formed between Thr862 and the ligand’s morpholine moiety. A new hydrogen bond is formed between the sulfonamide and Lys753. Hydrophobic interactions shift toward Val797, Ala751, Thr798, Ile752, Val734, and Arg71. The simulation with FGFR1 shows the formation of an extra hydrogen bond interaction with Glu486. VDW interaction shifted toward Gly487 and Gly485. The 180° flip of the ligand’s naphthalene moiety is coherent with the RMSD shift at around 13 ns. In FGFR2, a hydrogen bond is formed between Lys517 and the sulfonamine moiety in Z21. In IGF1R, the hydrogen bond with Lys1003 is shifted from the secondary aniline to the sulfonamine in Z21. In TrkA, an extra hydrogen bond is formed with Lys523. The conformational change at the morpholine–sulfonamine moiety is consistent with the RMSD plot of Z21 at 8 ns and 16 ns (Figure 2A). The same conformational change is seen in VEGFR1 that additionally forms a hydrogen bond between Glu878 and the nitrogen of the secondary aniline. In contrast to the TrkA complex, the conformational change of Z21 in the VEGFR1 complex occurred in the early equilibration phase. In FGFR4, the ligand remained stable throughout the 20 ns simulation with a small rotational shift of the morpholine heterocycle attributed to the prior equilibration step. The hydrophobic interactions shifted toward Cys552 and Glu551. In general, fluctuations in RMSD are consistent with the ratio between the lost interactions and the newly created interactions. Seven out of eight complexes show the formation of a hydrogen bond in the course of the simulation, of which six were with the sulfonamide moiety (Figure 2D).

#### 3.2.2. Compound Z88 with RTKs

The predicted binding energy after docking Z88 (neutral tautomer) into RTKs indicated the strongest interaction with INSR (−10.3 kcal/mol) (Figure 3A). Converged RMSD plots from the MD simulations for the six most stable complexes are shown in Figure 3B. The six most stable complexes after MD are shown superimposed in Figure 3C. Interaction plots of the individual complexes before and after MD are shown in Figure 3D.

In EGFR, a hydrogen bond is formed between Lys721 and O1 carbonyl oxygen of the substituted 2, 3-dihydrophtalazine-1,4-dione moiety. New hydrophobic interactions are formed with Gly700, Thr701, Glu722, Ala719, Ile720, Met742 and Arg817. With ERBB2, Phe864 donates a hydrogen bond to the O1 carbonyl oxygen of the dihydrophtalazine moiety and a new hydrophobic interaction is formed with Ala43. In FGFR2, the backbone nitrogen of Ala567 forms a strong a hydrogen bond formation with the O2 carbonyl oxygen of the 2, 3-dihydrophtalazine-1,4-dione moiety. Hydrophobic interactions are formed with Arg630, Ala643, Glu534, Met538, Phe645 and Glu565. In complex with IGF1R, a new hydrogen bond is formed between Arg673 backbone nitrogen and the carbonyl oxygen of the central tertiary amide linker together with three new hydrophobic interactions with Val573, Gly667 and Asn655. The complex with TrkA shows the formation of two new hydrogen bonds between the carbonyl oxygen of the central tertiary amide moiety with Lys861 and Leu1043. New hydrophobic interactions are formed with Thr1059, Thr877 and Cys1039. In VEGFR1, Z88 interacts hydrophobically with Asn1110, Val1033 and Cys1111, while forming a stable hydrogen bond between Met1052 and the O2 carbonyl oxygen of the dihydrophtalazine moiety.

#### 3.2.3. Compound AF3 with SKTs

The predicted binding energy after docking AF3 into STKs indicated the strongest interaction with the proto-oncogene kinase PIM1 (-12.2 kcal/mol) (Figure 4A). Converged RMSD plots from the MD simulations for the six most stable complexes are shown in Figure 4B. Superimposed complexes and 2D interaction plots of the individual complexes before and after MD are displayed in Figure 4C,D.

In AURKA, Lys162 contributes a hydrogen bond with the carbonyl oxygen of the ester linker. New hydrophobic interactions with Tyr212, Leu194, Leu210, Glu211, Asp274, Gln187 and Leu178 are formed. The large shift in interacting amino acids is consistent with the higher value and larger fluctuations in the RMSD values, as shown in the respective plot. In AURKB, a hydrogen bond is formed between Ala88 and the carbonyl oxygen within the chromenone moiety. Additionally, new hydrophobic interactions are formed with Lys85, Ala217 and Pro158. In complex with CHEK2, Thr367 donates a stable hydrogen bond to the carbonyl oxygen of the ester linker. Moreover, various hydrophobic interactions are seen with Leu226, Gly227, Ser228, Ala247, Ile274, Ile286 and Glu302. In complex with JNK1, Ile147, Ile110 and Ile32 interact hydrophobically. In this case, the large number of conserved interactions is consistent with the respective RMSD plot. In case of the ERK1 complex, a weak and rather rare hydrogen bond is formed with the ether oxygen of the ester linker together with hydrophobic interactions with Gly49 and Thr207. In complex with ERK2, AF3 binding is stabilized with four hydrogen bonds of which three are formed during the MD simulation. One hydrogen bond between the chromenone moiety and Asn154 is conserved. One new hydrogen bond is formed between Arg67 and the carbonyl oxygen of the ester linker and two new hydrogen bonds are formed between Arg191 and the oxygen of the anisole moiety. Three new hydrophobic interactions are formed with Asp149, Lys151 and Thr190. For PIM1, a new hydrogen bond is formed with Ser189. Gly45, Ser46, Gly47, Ile185 and Gly188 stabilize the complex through hydrophobic interactions.

#### 3.2.4. Compound F34 with STKs

The predicted binding energy after docking F34 into STKs indicated the strongest interaction with the proto-oncogene kinase PIM2 (−11.6 kcal/mol) (Figure 5A). Converged RMSD plots from the MD simulations for the six most stable complexes are shown in Figure 5B. Superimposed complexes and 2D interaction plots of the individual complexes before and after MD are displayed in Figure 5C,D.

In AURKA, a new hydrogen bond is formed between the carbonyl oxygen of the pyrimidinone moiety and Lys162. New hydrophobic interactions with Ala160, Leu164, Leu169, Glu211, and Glu260 are formed. In CHEK2, three hydrogen bonds are formed, of which two are new. Met304 forms a new hydrogen bond with the oxadiazolidine/oxadiazole moiety. Gly20 forms the second new hydrogen bond with the carbonyl oxygen of the amide linker that is also accepting the conserved hydrogen bond from Lys249. New hydrophobic interactions are formed with Val231. In ERK, a hydrogen bond is formed between Ser170 and the oxadiazolidine/oxadiazole moiety of the ligand. Hydrophobic interactions are established with Gly49, Tyr130, Lys131, and Cys183. In ERK2, a hydrogen bond is observed with Lys54. Additionally, two new hydrogen bonds are seen, of which one is between Asp169 and the nitrogen of the amide linker, and the second is between Lys55 and the oxadiazolidine/oxadiazole moiety. Asp167, Ala53, His66, and Asn114 interact hydrophobically. Finally, the plot for the PIM1 interaction with F34 shows only a shift from VDW interactions toward Glu135 and Leu177.

### 3.3. Estimated Total Binding Free Energy (MM-GBSA) Calculations

To further estimate the binding energetics of all four screened compounds, the AMBER mmgbsa module was used to calculate the binding free energy of each ligand with their respective targets. Due to their multi-target nature, the mean value of each compound was calculated per kinase subclass. In case of the receptor tyrosine kinases subclass, an average binding affinity of −35.62 (±3.29) kcal/mol and −41.88 (±4.83) kcal/mol was found for compounds Z88 and Z21, respectively (Table 1). This result suggests a more favorable interaction with Z21, with the highest binding affinity for EGFR (−48.59 kcal/mol). In the case of the serine tyrosine kinases subclass, an average binding affinity of −34.13 (±7.87) kcal/mol and −47.90 (±5.29) kcal/mol was found for compounds AF3 and F34, respectively. The more favorable mean interaction energy with F34 correlates with the in vitro findings. The strongest affinity of lead compound F34 was found in complex with the PIM1 kinase (−56.06 kcal/mol).

### 3.4. In Vitro Cytotoxicity

**Figure 6:**
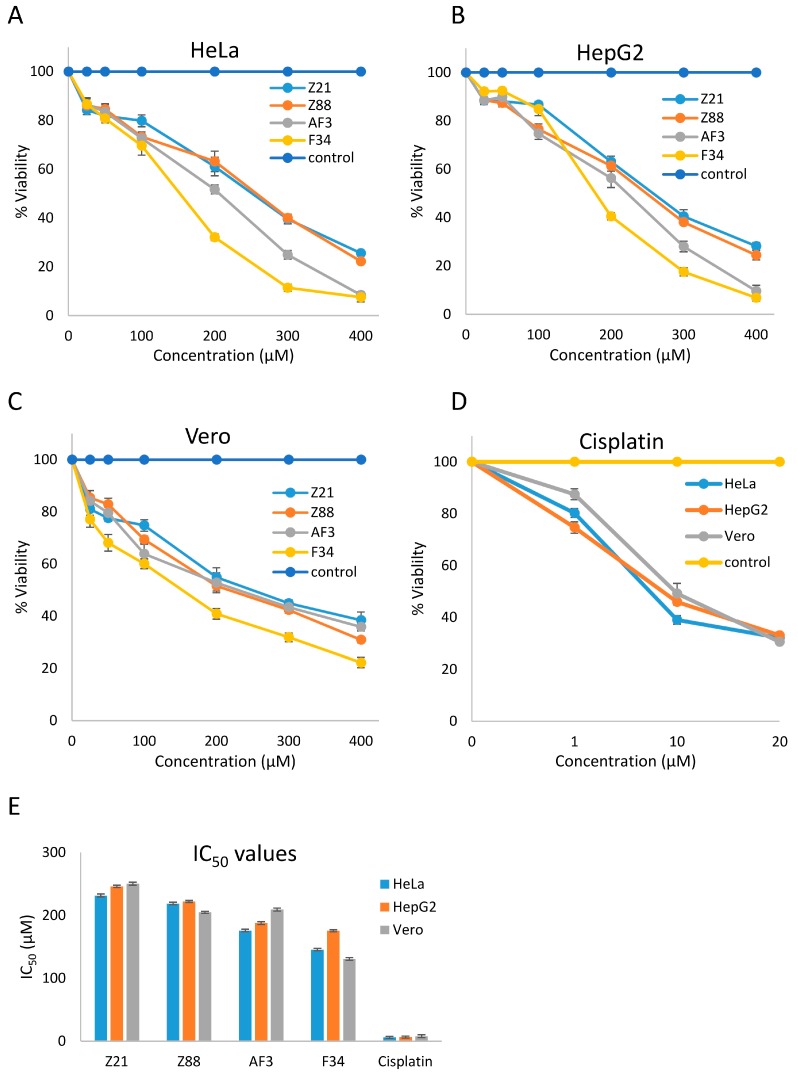
Activities of predicted STK and RTK inhibitors against human epithelial cervical cancer (HeLa), HepG2, and monkey epithelial kidney cancer (Vero) cell lines. (**A**) Dose–response curves of Z21, Z88, AF3, and F34 against HeLa cell line. Percentage viabilities of HeLa cells against concentration (µM) after 48 h of treatment. (**B**) Dose–response curves of Z21, Z88, AF3, and F34 against the HepG2 cell line. Percentage viabilities of HepG2 cells against concentration (µM) after 48 h of treatment. (**C**) Dose–response curves of Z21, Z88, AF3, and F34 against the Vero cell line. Percentage viabilities of Vero cells against concentration (µM) after 48 h of treatment. (**D**) Dose–response curves of cisplatin against HeLa, HepG2, and Vero cell lines. Percentage viabilities of different cell lines against concentration (µM) of cisplatin after 48 h of treatment. (**E**) Graphical representation of IC_50_ values of Z21, Z88, AF3, F34 and cisplatin after 48 h treatment against HeLa, HepG2 and Vero cell lines using cisplatin as a positive control.

These four compounds were subsequently tested for their cytotoxic effect on different cell lines. Cell viability assays on cancer cell lines HeLa and HepG2 and non-cancerous Vero cell line were performed using the lead compounds (Z21, Z88, F34, AF3) with doses ranging from 50 to 400 µM. Cisplatin was used as a positive control at dose levels of 1 µM, 10 µM, and 20 µM. Dose–response curves of all four inhibitors against the three cell lines show the moderate sensitivity of all cell line toward these inhibitors (Figure 6). The IC_50_ values for these four compounds ranged between 231.44 to 145.46 µM in the HeLa cell line, 245.96 to 175.48 µM in HepG2 cell line, and 250.24 to 130.52 µM in Vero lines (Table 2). F34 proved to be the most potent against HeLa, HepG2, and Vero cell lines with IC_50_ values of 145.46 µM, 175.48 µM, and 130.52 µM, respectively.

## 4. Discussion

The overexpression and upregulation of protein kinases have been associated with a wide variety of carcinomas [1,135,136,137,138,139]. Anti-cancer agents with multiple targets involved in different signaling pathways have been shown to be more effective against cancers than agents targeting single pathways [140]. Therefore, targeting multiple kinases can exponentially increase the success rates of anti-cancer therapies [20,23,84,141]. Several successful studies have been carried out to discover anti-cancer agents with broad polypharmacological effect against multiple RTKs or STKs. For example, sunitinib and sorafenib are orally administered multi-kinase (small molecule) inhibitors that exhibit potent antitumor activity by targeting several protein kinases, including VEGFR, platelet-derived growth factor receptor (PDGFR), ERK, proto-oncogene kinases RAF and KIT, and fms-like tyrosine kinase 3 (FLT3) [142,143,144]. Well-known multi-kinase inhibitors, dasatinib and lapatinib, are reported to inhibit Abelson murine leukemia viral oncogene homolog 1 (ABL) tyrosine kinase, EGFR, and ERBB2 [75,76] while molecular targets for imatinib include KIT, PDGFR, and ABL [145].

Growth and proliferation-related pathways in cancer cells require multiple types of STKs and RTKs [135,136,140]. We identified four cytotoxic compounds as putative STK and/or RTK inhibitors that can serve as starting points for the development of novel (multi-target) kinase inhibitors as anti-cancer drugs. An extensive SBVS protocol was carried out by docking of a large compound library against representative STK and RTK protein kinases. The stability of their predicted binding poses in the studied STKs and RTKs was assessed by MD simulations, which displayed a stable interaction in all of the simulated kinase/ligand complexes. The top virtual hits against RTKs (Z21 and Z88) and STKs (AF3 and F34) were tested against various cell lines to measure their cytotoxicity.

The extensive investigation of the molecular interactions after MD simulation revealed prominent consistency with respect to many experimentally resolved inhibitor binding poses that have been reported and are available as co-crystals at the PDB. For example, residues Thr766 and Lys721 of EGFR that are predicted to form hydrophobic interactions with Z21 and Z88 (Figure 2D and Figure 3D) were reported by Palmer et al. [146] to interact within ~3-Å distance with the crystallized inhibitor. In ERBB2 complexes, as defined by Ishikawa et al. [147], Thr862 and Asp863 were found to improve the specificity for pyrimidine derivatives in the hinge region of the ATP-binding pocket. In FGFR2, residues participating in hydrophobic interactions (Leu633, Phe644, Ala567, and Leu487, Val495, and Lys517) are reported to be involved in forming the A-loop and glycine-rich loop, respectively (Figure 2D and Figure 3D) and take part in the ATP-binding cleft involved in its complex mechanism of inhibition [148]. The interaction analysis of the FGFR2-Z21/Z88 complexes showed some of the same interacting residues as those reported for a co-crystallized inhibitor, ARQ 069 [149]. Similarly, STK complexes with AF3 and F34 show similar interacting residues as found in the crystal complexes of various kinase inhibitors. For example, the X-ray complex of AURKA with SAR156497 [150] showed Lys162 as an H-bond forming residue. This interaction is conserved in both AF3 and F34 complexes, together with consistent hydrophobic contacts (Glu211 and Tyr212). CHEK-2 complexed with AF3 and F34 shares common interacting residues; Lys249, Met304, Leu226, and Leu354 rendering ligand NSC109555 a notably potent and selective inhibitor against CHEK2 [151]. JNK1 complexed with AF3 and F34 indicated several favorable interacting residues such as Asp169, which forms a hydrogen bond in both complexes together with Lys55, Met111, and Glu73. These three participate in hydrophobic interactions, as observed in the crystal structure of JNK1 bound with AX13587 [152]. Similarly, post-docking analysis of the ERK1–F34 complex confirmed the presence of a hydrogen bond between Ser170 and F34 together with hydrophobic contacts formed by Asp184, Val56, Leu172, and Met125, which were suggested as favorable interaction sites for ERK inhibitors in the crystallographic study by Kinoshita et al. [153].

All four test compounds have chemically distinct structures compared to reported serine/threonine and receptor tyrosine kinase inhibitors. Nevertheless, some degrees of analogy between these compounds and certain reported kinase inhibitors can be seen. Zinc, ChEMBL, and drugbank libraries were explored to identify compounds with the related type of activities. According to this literature survey, the related analogues of our compounds show involvement in serine/threonine and receptor tyrosine kinase pathways.

N-(4-chloro-3-(morpholinosulfonyl)phenyl)-1-naphthamide, an analogue of Z21, has been reported to have IC_50_ in the nanomolar range against the cannabinoid CB1 receptor, which is a cell membrane receptor in the G protein-coupled receptor family [154]. Via signal transduction, it has a downstream role with protein kinase A and C, ERK, JNK, c-Jun, c-Fos, Raf-1, p38 and other kinases [155].

An analogue of Z88 (benzo-1,4-dioxane containing derivative of 2,3-dihydrophthalazine-1,4-dione) is reported to inhibit human tyrosyl-DNA phosphodiesterase 1, which is involved in the catalysis of 3′-phosphotyrosyl bond hydrolysis [156].

AF3 is analogous to plant flavonoids myricetin and quercetin containing 3,7-dihydroxy-4H-chromen-4-one residue. Myricetin inhibits protein kinase C, MAPK, c-Jun N-terminal kinase (PKC, MAPK, and Jnk) and tyrosine protein kinase JAK1. JAK1 is a non-receptor type tyrosine kinase involved in the signaling pathway of IFN-alpha/beta/gamma and acts as a kinase partner for the interleukin (IL)-2 receptor [157].

Myricetin has serine/threonine kinase activity toward phosphatidylinositol 4,5-bisphosphate 3-kinase and catalytic activity toward subunit gamma isoform [158]. Quercetin is an inhibitor of serine/threonine protein kinase PIM1 via transcription factor binding. PIM1 is a proto-oncogene with serine/threonine kinase activity involved in cell proliferation and cell survival, and thus providing a selective advantage in tumorigenesis. Additionally, protein serine/threonine kinase activity has been shown through binding with serine/threonine protein kinase 17B. Moreover, it can phosphorylate myosin light chains, and acts as a positive regulator of apoptosis [159]. Quercetin has also been found to possess protein serine/threonine kinase activity via the inhibition of casein kinase 2 subunit alpha, which stops the phosphorylation of proteins containing acidic residues [160].

F34 is analogous to Relugolix and Sufugolix, incorporating thieno[2,3-d]pyrimidin-4(3H)-one as a common scaffold. Both of these compounds act as an antagonist for the gonadotropin-releasing hormone, which is being clinically studied for prostate cancer, uterine leiomyoma, and endometriosis [161,162].

Different FDA-approved kinase inhibitors have IC_50_ values in the nanomolar range against tested cell lines (vandetanib (EGFR, VEGFR, RET, Tie-2, FGFR1; IC_50_ <500 nM), sunitinib (VEGFR2, PDGFRβ, KIT, RET, CSF1R, FLT3; IC_50_ <100 nM), sorafenib (VEGFR, PDGFR, B-RAF, MEK, ERK; IC_50_ <100 nM), regorafenib (TIE2, PDGFR, RET, KIT, B-RAF; IC_50_ <25 nM), ponatinib (BCR-ABL, PDGFRα, SRC, KIT, FGFR, VEGFR; IC_50_ <6 nM), pazopanib (PDGFR, VEGFR; IC_50_ <150 nM), nilotinib (BCR-ABL, KIT, LCK, EPHA3, 8, DDR1, 2; IC_50_ <30 nM), imatinib (ABL, KIT, PDGFR; IC_50_ <0.6 nM), dasatinib (BCR-ABL, SRC, KIT, PDGFR, EPH, CSK; IC_50_ <10 nM), cabozantinib (VEGF, RET, MET, NTRKB, TIE2, AXL; IC_50_ <15 nM) and axitinib (VEGFRs, PDGFRs, KIT; IC_50_ <1.7 nM)). The newly identified, potentially multi-targeting STK and RTK inhibitors (Z21, Z88, AF3, and F34) presented IC_50_ values of less than 251 μM in HeLa, HepG2, and Vero cell lines with cisplatin as a positive control. F34 is presented as the most viable hit, supported by the MD analysis and in vitro results with IC_50_ values of 145.46 μM, 175.48 μM, and 130.52 μM in HeLa, HepG2, and Vero cell lines, respectively. Given the presence of both RTKs and STKs in all of the cell lines, F34, a hit compound against the studied STKs, was cross-docked into the RTK crystallized apo structure using Glide. Z88, Z21, and AF3 were docked in parallel to compare their predicted binding affinities with F34 (Supplementary Table S4). The F34 scaffold showed top-ranked binding affinity in seven out of 13 calculated complexes. Therefore, it has potential as a promising starting scaffold for further in vitro SAR studies with specific oncogenic kinases. Moreover, synergistic effects can be determined with known STK and RTK inhibitors.

## 5. Conclusions

A combination of computational methods helps us not only to increase search space but also to decrease experimental costs by aiding in the selection of potential scaffolds for experimental assays. Generally, most computational tools are experimentally validated. In this study, we determined the network of interactions established between the ligands and each target kinase and compared this to multiple co-crystalized inhibitors, thus aiming at retrospective validation. However, there is a trade-off between the highly specific nature of individual enzymes and the multi-target concept of the presented work. A single scaffold per kinase class, as presented, is unlikely to bind strongly to each individual enzyme. The resulting suboptimal whole-cell activities could be attributed to the more general nature of the scaffold. Therefore, these scaffolds provide a strong starting point for individual targeting of STKs and RTKs with further enzymatic assays that are essential to substantiate the multi-target hypothesis. Furthermore, the binding affinity results from kinase assays can be iteratively included to significantly potentiate the presented virtual screening workflow.

## Figures and Tables

**Table 1:** Total binding free energy of top four hits in complex with respective kinase targets computed by the AMBER molecular mechanics–generalized Born surface area (MM-GBSA) method (kcal/mol). EGFR: epidermal growth factor receptor; FGFR(1,2,4): fibroblast growth factor receptor 1, 2, 4; VEGFR1: vascular endothelial growth factor receptor 1; IGF1R: insulin-like growth factor 1 receptor; TrkA: tropomyosin receptor kinase A.

RTK Targets	Compound	ΔG_tol_	STK Targets	Compound	ΔG_tol_
EGFR	Z88	−33.58	AURKA	F34	−50.76
	Z21	−48.59		AF3	−41.11
FGFR1	Z88	−37.16	AURKB	F34	−45.18
	Z21	−36.88		AF3	−39.76
FGFR2	Z88	−41.01	ERK1	F34	−52.65
	Z21	−42.94		AF3	−17.99
FGFR4	Z88	−36.76	ERK2	F34	−42.03
	Z21	−33.37		AF3	−38.65
VEGFR1	Z88	−38.39	JNK1	F34	−45.5
	Z21	−41.33		AF3	−33.23
ERBB2	Z88	−33.93	CHEK2	F34	−43.11
	Z21	−45.93		AF3	−36.27
IGF1R	Z88	−30.82	PIM1	F34	−56.06
	Z21	−42.64		AF3	−31.87
TrkA	Z88	−33.33			
	Z21	−43.39			

**Table 2:** IC_50_ (µM) values (mean ± S.D) of Z21, Z88, AF3, F34, and cisplatin (positive control) after 48 h of treatment against HeLa, HepG2, and Vero cell lines.

	Z21	Z88	AF3	F34	Cisplatin
HeLa	231.44 ± 2.35	218.68 ± 2.04	175.69 ± 2.12	145.46 ± 2.06	6.02 ± 1.59
HepG2	245.96 ± 1.93	222.05 ± 1.68	187.54 ± 2.31	175.48 ± 1.66	6.41 ± 1.54
Vero	250.24 ± 2.34	204.78 ± 1.78	209.16 ± 2.43	130.52 ± 2.3	7.99 ± 2.38

## Supplementary Materials

The following are available online at https://www.mdpi.com/2218-273X/9/4/124/s1. Table S1: Receptor-Tyrosine Kinases data set; Table S2: Serine-Threonine Kinases data set; Table S3: Detailed physicochemical, drug-likeness, ADMET and medicinal chemistry friendliness evaluation of top four hit compounds; Table S4: Glide docking results for RTKs; Figure S1: Multiple sequence alignment of some of the kinases used in the study; Figure S2: Structural insight in terms of superimposition of RTKs (above panel) and STKs (below panel).
