# Supplementary material for: Inhibition of Oncogenic Kinases: An In Vitro Validated Computational Approach Identified Potential Multi-Target Anticancer Compounds
Source: Biomolecules. 2019 Mar 28;9(4):124. doi: 10.3390/biom9040124 (PMC6523505; doi:10.3390/biom9040124)
Supplement: Supplementary file 1 [file biomolecules-09-00124-s001.pdf]

Inhibition of Oncogenic Kinases: An In Vitro Validated Computational Approach Identified Potential Multi-Target Anticancer Compounds

Nazia Ikram[1], Muhammad Usman Mirza[2,3*], Michiel Vanmeert[3], Matheus Froeyen[3], Outi M. H. Salo-Ahen[4,5], Muhammad Tahir[2], Aamer Qazi[2], Sarfraz Ahmad[6,7]

[1] Institute of Molecular Biology and Biotechnology, The University of Lahore, 54000 Lahore, Pakistan; naxiaikram@gmail.com (N.I)

[2] Centre for Research in Molecular Medicine, The University of Lahore, 54000 Lahore, Pakistan; muhammad.tahir@imbb.uol.edu.pk (M.T.); aamer.qazi@imbb.uol.edu.pk (A.Q.)

[3] Department of Pharmaceutical and Pharmacological Sciences, Rega Institute for Medical Research, Medicinal Chemistry, University of Leuven, B-3000 Leuven, Belgium; michiel.vanmeert@kuleuven.be (M.V.); mathy.froeyen@kuleuven.be (M.F.)

[4] Structural Bioinformatics Laboratory, Faculty of Science and Engineering, Biochemistry, Åbo Akademi University, FI-20520 Turku, Finland; outi.salo-ahen@abo.fi (O.M.H.S.-A.)

[5] Pharmaceutical Sciences Laboratory, Faculty of Science and Engineering, Pharmacy, Åbo Akademi University, FI-20520 Turku, Finland

[6] Institute of Pharmaceutical Sciences, Riphah University, 54000 Lahore, Pakistan; sarfraz.ahmad@riphah.edu.pk (S.A)

[7] Department of Chemistry, Faculty of Sciences, University Malaya, 59100, Kuala Lumpur, Malaysia

*Corresponding author

Muhammad Usman Mirza, Department of Pharmaceutical and Pharmacological Sciences, Rega Institute for Medical Research, Medicinal Chemistry, University of Leuven, Leuven B-3000, Belgium.
Tel.: +32-48-539-9542
E-mail: muhammadusman.mirza@kuleuven.be (M.U.M)

Table S1: Receptor-Tyrosine Kinases data set

Aliases	Uniprot ID	PI/MW	Protein Kinase domain position (Length)	PDB ID.Chain	Resolution (Å)	R-Value Free	Bound inhibitor	Role in Cancer	Reference
EGFR, ERBB, ERBB1, HER1, NISBD2, PIG61, mENA	P00533	6.26/ 134277.4	712-979 (268)	1M17.A	2.6	0.295	erlotinib	Overexpressed in several cancers	[1,2]
ERBB2, CD340, HER-2, HER-2/neu, HER2, MLN 19, NEU, NGL	P04626	5.58/ 137910.5	729-987 (268)	3RCD.A	3.21	0.294	TAK-285	Overexpressed in breast cancer, ovarian, stomach, adenocarcinoma of the lung, endometrial carcinoma	[3,4]
IGF1R, CD221, IGFR, JTK13, Insulin-like growth factor 1 receptor beta chain	P08069	5.58/ 154793.0	999-1274 (276)	3D94.A	2.3	0.236	PQIP	Overexpressed in several cancers	[5,6]
INSR, CD220, HHF5, insulin receptor	P06213	5.83/ 156332.8	1023-1298 (276)	3ETA.A	2.6	0.232	pyrrolopyridine	Ooverexpression of INSR increases tumor growth, proliferation, colony formation, migration, invasion and angiogenesis	[7,8]
FLT3, CD135, FLK2, STK1, FMS-like tyrosine kinase 3	P36888	5.48/ 112903.4	610-943 (334)	4XUF.A	3.2	0.320	quizartinib (AC220)	Overexpressed in several cancers	[1,2,9]
KIT, C-Kit, CD117, PBT, SCFR, Mast/stem cell growth factor receptor	P10721	6.54/ 109864.5	589-937 (349)	4U0I.A	2	0.239	ponatinib	Overexpression leading to overactivation of various signalling pathways	[10,11]
FGFR1, BFGFR, CD331, CEK, FGFBR, FGFR-1, FLG, FLT-2, FLT2, HBGFR, HH2, HRTFDS, KAL2, N-SAM, OGD, bFGF-R-1, ECCL, fibroblast growth factor receptor 1	P11362	5.82/ 91867.74	478-767 (290)	5A46.A	2.63	0.246	dovitinib	Overexpression leading to overactivation of various signalling pathways	[12,13]
FGFR2, BBDS, BEK, BFR-1, CD332, CEK3, CFD1, ECT1, JWS, K-SAM, KGFR, TK14, TK25, fibroblast growth factor receptor 2	P21802	5.61/ 92025.01	481-770 (290)	3RI1.A	2.1	0.247	ARQ 069	Pancreatic, kidney, lung, ovarian cancer	[13,14]

FGF receptor 4, FGFR4	P22455	6.36/ 87954.45	467 – 755 (289)	4QRC.A	1.901	0.207	ponatinib	Pancreatic, kidney, lung, ovarian cancer	[13,14]
FLT1, FLT, FLT-1, VEGFR1, Vascular endothelial growth factor receptor 1	P17948	8.66/ 150768.6	827-1158 (332)	3HNG.A	2.7	0.260	benzamide	Upregulate/transactivate various pathways	[11,13,15]
KDR, CD309, FLK1, VEGFR2, Vascular endothelial growth factor receptor 2	P35968	5.60/ 151526.8	834-1162 (329)	2QU6.A	2.1	0.272	benzoxazole	Upregulate/transactivate various pathways	[13,16,17]
MET, MET proto-oncogene, receptor tyrosine kinase, AUTS9, HGFR, RCCP2, c-Met, DFNB97, OSFD	P08581	7.02/ 155541.3	1078-1345(268)	2RFS.A	2.2	0.262	SU11274	Upregulate/transactivate various pathways	[18–20]
NTRK1, MTC, TRK, TRK1, TRKA, Trk-A, p140-TrkA, High affinity nerve growth factor receptor	P04629	6.17/ 87497.14	510-781 (272)	4YPS.A	2.1	0.257	4-{6-[(3R)-3-(3-fluorophenyl)morpho-lin-4-yl]imidazo[1,2-b]pyridazin-3-yl}benzonitrile	Prostate, gastric cancer and melanoma	[16,21,22]

Table S2: Serine-Threonine Kinases data set

Aliases	Uniprot ID	PI/MW	Protein Kinase domain position (Length)	PDB ID.Chain	Resolution (Å)	R-Value Free	Bound inhibitor	Role in Cancer	Reference
CK2 alpha, CSNK2A1, Csnk2a1, Csnk2a1-rs4, CK2A1, CKII, CSNK2A3, Casein kinase II subunit alpha	P68400	7.29/ 45114.80	39-324 (286)	3PE1.A	1.6	0.203	CX-4945	Overexpressed in prostatic carcinoma	[23,24]
AURKA, AIK, ARK1, AURA, AURORA2, BTAK, PPP1R47, STK15, STK6, STK7, AURORA2 Kinase domain	O14965	9.45/ 45809.36	133-383 (251)	4UZH.A	2.0	0.235	SAR156497	Overexpressed in Breast and prostate cancer	[25,26]
AURKB, AIK2, AIM-1, AIM1, ARK2, AurB, IPL1, PPP1R48, STK12, STK5, AURORA kinase B	Q96GD4	9.36/ 39310.54	77-327 (251)	4AF3.A	2.75	0.264	VX-680	Overexpressed in several cancers	[27]
CHEK1, CHK1, checkpoint kinase 1	O14757	8.50/ 54433.57	9-265 (257)	4QYH.A	1.9	0.235	diazacarbazole GNE-783	Overexpressed innumerous tumors	[28,29]
CHEK2, CDS1, CHK2, HuCds1, LFS2, PP1425, checkpoint kinase 2	O96017	5.65/ 60914.84	220-486(267)	2W0J.A	2.05	0.246	NSC 109555	Breast cancer	[30]
PLK1, PLK, STPK13, polo like kinase 1	P53350	9.09/ 68254.78	53-305 (253)	2RKU.A	1.95	0.241	BI2536	Colorectal, bladder, prostate and nasopharyngeal cancer	[31–33]
PLK4, SAK, STK18, MCCRP2, polo like kinase 4	O00444	8.79/ 108971.95	12-265 (254)	4JXF.A	2.4	0.268	(1R,2S)-2-{3-[(E)-2-{4-[(dimethylamino)methyl]phenyl}ethenyl]-2H-indazol-6-yl}-5'-	Bladder, liver cancer	[34,35]

							methoxyspiro[cyclo-propane-1,3'-indol]-2'(1'H)-one		
PIM1, PIM, Pim-1 proto-oncogene	P11309	6.52/ 45412.37	129-381 (253)	2C3I.B	1.9	0.219	imidazopyridazin I	Bladder, prostate cancer, adeno-carcinoma	[36,37]
PIM2, Serine/threonine-protein kinase pim-2	Q9P1W9	5.58/ 34190.37	32-286 (255)	4X7Q.A	2.33	0.258	2-(2,6-difluorophenyl)-N-{4-[(3S)-pyrrolidin-3-yloxy]pyridin-3-yl}-1,3-thiazole-4-carboxamide	Prostate	[38–40]
MAPK3, ERK-1, ERK1, ERT2, HS44KDAP, HUMKER1A, P44ERK1, P44MAPK, PRKM3, p44-ERK1, p44-MAPK, mitogen-activated protein kinase 3	P27361	6.28/ 43135.57	42 - 330 (289)	2ZOQ.A, B	2.39	0.267	5-iodotubercidin	Overexpression leading to overactivation of various signalling pathways	[41–44]
MAPK1, ERK, ERK-2, ERK2, ERT1, MAPK2, P42MAPK, PRKM1, PRKM2, p38, p40, p41, p41mapk,	P28482	6.50/ 41389.71	25 - 313 (289)	1TVO.A	2.5	0.272	FR180204	Overexpression leading to overactivation of various signalling pathways	[43,45]
MAPK8, Mapk8, AI849689, JNK, JNK1, Prkm8, SAPK1, JNK-46, JNK1A2, JNK21B1/2, SAPK1c, mitogen-activated protein kinase 8	P45983	6.43/ 48295.56	26-321 (296)	4L7F.A	1.95	0.198	AX13587	Pancreatic, Kidney, Lung, ovarian cancer	[46–48]
MAPK9, JNK-55, SAPK1a, PRKM9, JNK2, mitogen-	P45984	5.41/ 48139.06	26-321 (296)	3NPC.A	2.35	0.254	BIRB796	Upregulate/transactivate various pathways	[49]

activated protein kinase 9									
MAPK10, JNK3, JNK3A, PRKM10, SAPK1b, p493F12, p54bSAPK, mitogen-activated protein kinase 10	P53779	6.33/ 52585.44	64-359 (296)	1PMU.A	2.7	0.285	phenantroline	Upregulate/transactivate various pathways	[44,46,50]
MAPK14, CSBP, CSBP1, CSBP2, CSPB1, SAPK2a, MXI2, MAX-interacting protein 2, MAPK p38 alpha, mitogen-activated protein kinase 14	Q16539	5.48/ 41293.29	24-308 (285)	1A9U.A	2.5	0.240	SB203580	Upregulate/transactivate various pathways	[44,46,51]
BRAF, B-RAF1, BRAF1, NS7, RAFB1, B-Raf, B-Raf proto-oncogene, serine/threonine kinase	P15056	7.29/ 84436.89	457-717 (261)	1UWH.A	2.95	0.257	BAY439006 (sorafenib)	Prostate, gastric cancer and melanoma	[52,53]
CDK6, MCPH12, PLSTIRE, cyclin-dependent kinase 6	Q00534	5.39/ 28637.45	13-300 (287)	2EUF.B	3	0.306	PD0332991	Overexpressed in lymphoma, leukemia,medulloblastoma and melanoma	[34,54]
CDK1, CDC2, CDC28A, P34CDC2, cyclin-dependent kinase 1	P06493	8.38/ 34095.45	4-287 (284)	4Y72.A	2.3	0.252	{[(2,6-difluorophenyl)carbonyl]amino}-N-(4-fluorophenyl)-1H-pyrazole-3-carboxamide	Breast cancer and involved in cancer cell cycles	[34,55,56]

Table S3: Detailed physicochemical, drug-likeness, ADMET and medicinal chemistry friendliness evaluation of top four hit compounds.

Top Hits	RTKs		STKs	
	Z217168138 (Z21)	Z88445222 (Z88)	AF-399/40714045 (AF3)	F3411-7101 (F34)
**Molecular Property**				
Mass	382.47	421.44	438.4262	491.9494
logP	4.5645	2.1527	5.9663	5.1569
H-bond acceptors	5	8	6	8
H-bond donors	1	1	0	1
Rotatable bonds	5	5	6	6
PSA	67.02	93.63	74.97	131.15
Molecular Refractivity (MR)	111.03	119.18	124.3985	132.6457
Atoms	49	54	51	52
Rings	4	5	5	5
Heavy atoms	27	31	33	34
Hydrogen atoms	22	23	18	18
Heteroatoms	6	8	6	10
N/O atoms	5	8	6	8
Inorganic atoms	0	0	0	0
Halogen atoms	0	0	0	1
Chiral centers	0	1	0	0
R/S chiral centers	0	1	0	0
Unknown chiral centers	0	0	0	0
Undefined chiral centers	0	0	0	0
Stereo double bonds	0	0	0	0
Cis/trans stereo double bonds	0	0	0	0
Unknown stereo double bonds	0	0	0	0
Undefined stereo double bonds	0	0	0	0
**Water Solubility**				

Log S	-4.47	-3.37	-6.24	-6.05
Water solubility	-3.63	-3.17	-3.35	-3.73
Solubility class	Moderately soluble	Soluble	Poorly soluble	Poorly soluble
**Druglikeness**				
Lipinski	Yes; 0 violation	Yes; 0 violation	Yes; 0 violation	Yes; 0 violation
Ghose filter	Yes	Yes	No; 1 violation: WLOGP>5.6	No; 1 violation: MW>480
Veber (GSK) filter	Yes	Yes	Yes	Yes
Egan (Pharmacia) filter	Yes	Yes	No; 1 violation: WLOGP>5.88	Yes
Muegge (Bayer) filter	Yes	Yes	No; 1 violation: XLOGP3>5	Yes
Abbott Bioavailability score	0.55	0.55	0.55	0.55
**Medicinal Chemistry (Friendly)**				
PAINS	0 alert	0 alert	0 alert	0 alert
Brenk structural alert	0 alert	0 alert	0 alert	0 alert
**ADMET**				
**A (Absorption)**				
Human Intestinal Absorption (HIA)	+	+	+	+
Human oral bioavailability (HOB)	-	-	+	-
Caco-2 permeability	-	-	-	-
**D (Distribution)**				
Plasma protein binding	1.215431452	1.188551426	1.057348371	1.228363633
P-glycoprotein inhibitor	+	+	+	+
P-glycoprotein substrate	-	-	-	-
Blood Brain Barrier (BBB)	+	+	-	+
**M (Metabolism)**				
CYP450 Inhibitors and Substrates				
CYP1A2 inhibition	-	-	+	-
CYP2C19 inhibition	+	-	-	-
CYP2C9 inhibition	-	+	-	+

CYP2C9 substrate	-	-	-	-
CYP2D6 inhibition	-	-	-	-
CYP2D6 substrate	+	-	-	-
CYP3A4 inhibition	-	-	-	-
CYP3A4 substrate	+	+	+	+
Pharmacokinetcs transporters				
BCRP inhibitor	-	-	+	-
BSEP inhibitor	+	+	+	+
OCT1 inhibitor	-	-	-	-
OCT2 inhibitor	-	+	-	-
MATE1 inhibitor	-	-	-	-
**E (Excretion)**				
Renal Organic Cation Transporter	-	-	-	-
**T (Toxicity)**				
Organ Toxicity				
Acute Oral Toxicity	III	III	III	III
Eye corrosion	-	-	-	-
Eye irritation	-	-	-	-
Genomic Toxicity				
Ames mutagenesis	+	+	+	-
Carcinogenicity	-	-	-	-
Micronucleus assay	+	+	+	+
Eco-toxicity				
Biodegradation	-	-	-	-

PSA, Polar surface accessibility; PAINS, Pan Assay Interference Compounds; CYP450, Cytochrome450; BCRP, Breast Cancer Resistance Protein; BSEP, human bile salt export pump; OCT, Organic cation transporter; MATE, Human multidrug and toxin extrusion transporter; Ghose filter (160 ≤ MW ≤ 480, -0.4 ≤ logP ≤ 5.6, 40 ≤ MR ≤ 130, 20 ≤ atoms ≤ 70); Veber filter (Rotatable bonds ≤ 10, PSA ≤ 140); Egan filter (logP ≤ 5.88, PSA ≤ 131.6); Muegge filter (200 ≤ MW ≤ 600, -2 ≤ logP ≤ 5, PSA ≤ 150, No. rings ≤ 7, No. carbon > 4, No. heteroatoms > 1, No. rotatable bonds ≤ 15, H-Bonds acceptor ≤ 10, H-Bonds donors ≤ 5).

Table S4: Glide docking results for RTKs

Compound	Prime/MMGBSA energy (kcal/mol)	Glide XP score (kcal/mol)	AutoDock Vina binding energy (kcal/mol)
EGFR (PDB ID: 1M17)			
Co-crystallized ligand	-50.919	N/A[a]	N/A
Re-docked co-crystallized ligand	-60.770	-9.007	-7.2
F34	-64.339	-8.592	-10.4
Z21	-45.699	-7.564	-8.9
Z88[b] (neutral OH/neutral NH)	-41.814/-35.470	-4.790/-5.073	-9.8
AF3	-38.691	-6.418	-4.9
ERBB2 (PDB ID: 3RCD)			
Co-crystallized ligand	-67.834	N/A	N/A
Re-docked co-crystallized ligand	-68.486	-11.488	-9.6
Z21	-59.482	-7.793	-9.7
Z88 (neutral OH/neutral NH)	-56.629/-41.531	-7.065/-8.731	-9.8
AF3	-45.256	-5.058	-10.2
F34	-55.242	-7.063	-10.7
IGF1R (PDB ID: 3D94)			
Co-crystallized ligand	-90.779	N/A	N/A
Re-docked co-crystallized ligand	-88.366	-14.703	-9.3
AF3	-74.078	-10.772	-10.7
F34	-66.983	-8.017	-10.8
Z88 (neutral OH/neutral NH)	-63.757/-49.491	-10.781/-12.056	-9.5
Z21	-58.766	-7.147	-9.9

Table S4: Glide docking results (continued)

Compound	Prime/MMGBSA energy (kcal/mol)	Glide XP score	AutoDock Vina binding energy (kcal/mol)
INSR (PDB ID: 3ETA)			
Co-crystallized ligand	-110.06	N/A	N/A
Re-docked co-crystallized ligand	-108.01	-13.533	-9.7
F34	-81.399	-10.738	-9.9
AF3	-49.882	-8.904	-9.8
Z21	-48.222	-8.374	-10.5
Z88 (neutral OH/neutral NH)	-45.452/-57.680	-8.006/-12.086	-10.3
FLT3 (PDB ID: 4XUF)			
Co-crystallized ligand	-92.767	N/A	N/A
Re-docked co-crystallized ligand	-86.796	-12.892	-8.9
F34	-82.045	-9.960	-10.6
AF3	-58.398	-10.280	-10.1
Z21	-48.325	-4.165	-9.1
Z88 (neutral OH/neutral NH)	-47.562/-6.004	-8.790/-5.090	-9.7
KIT (PDB ID: 4U0I)			
Co-crystallized ligand	-90.108	N/A	N/A
Re-docked co-crystallized ligand	-93.700	-13.796	-9.1
AF3	-70.03	-9.331	-8.6
Z21	-59.116	-8.783	-9.8
F34	-77.091	-8.789	-11.2
Z88 (neutral OH/neutral NH)	-48.597/-44.056	-8.134/-5.534	-9.3

Table S4: Glide docking results (continued)

Compound	Prime/MMGBSA energy (kcal/mol)	Glide XP score	AutoDock Vina binding score
FGFR1 (PDB ID: 5A46)			
Co-crystallized ligand	-51.126	N/A	N/A
Re-docked co-crystallized ligand	-49.887	-8.380	-8.5
Z88 (neutral OH/neutral NH)	-54.729/-35.388	-5.089/-7.710	-9.2
AF3	-54.196	-3.071	-9.1
F34	-52.863	-5.567	-9
Z21	-34.720	-4.231	-8.9
FGFR2 (PDB ID: 3RI1)			
Co-crystallized ligand	-56.099	N/A	N/A
Re-docked co-crystallized ligand	-56.095	-9.808	-8.4
AF3	-52.831	-7.249	-9.1
F34	-49.989	-5.462	-9.6
Z88 (neutral OH/neutral NH)	-46.595/-47.949	-5.998/-7.489	-9.7
Z21	-38.653	-6.212	-9.4
FGFR4 (PDB ID: 4QRC)			
Co-crystallized ligand	-70.029	N/A	N/A
Re-docked co-crystallized ligand	-82.974	-8.761	-8.3
F34	-61.277	-5.251	-10.7
Z88 (neutral OH/neutral NH)	-55.254/-31.803	-6.967/-7.184	-9.5
AF3	-37.437	-3.512	-9.7
Z21	-31.727	-7.887	-9.4

Table S4: Glide docking results (continued)

Compound	Prime/MMGBSA energy (kcal/mol)	Glide XP score	AutoDock Vina binding score
	VEGFR1 (PDB ID: 3HNG)		
Co-crystallized ligand	-85.492	N/A	N/A
Re-docked co-crystallized ligand	-84.998	-13.378	-9.1
F34	-68.387	-7.717	-7.3
AF3	-62.088	-8.684	-11.2
Z21	-54.304	-9.428	-10.7
Z88 (neutral OH/neutral NH)	-41.694/-40.015	-8.308/-7.749	-9.2
	VGFR2 (PDB ID: 2QU6)		
Co-crystallized ligand	-91.169	N/A	N/A
Re-docked co-crystallized ligand	-92.168	-13.76	-9.2
F34	-69.320	-9.489	-7.7
AF3	-63.035	-9.890	-9
Z21	-51.446	-7.732	-9.5
Z88 (neutral OH/neutral NH)	-41.699/-24.507	-7.583/-3.267	-9.4
	c-MET (PDB ID: 2RFS)		
Co-crystallized ligand	-62.922	N/A	N/A
Re-docked co-crystallized ligand	-62.990[c]	-6.740	-8.3
Z88 (neutral OH/neutral NH)	-62.530/-61.323	-9.922/-8.794	-9.9
AF3	-59.052	-6.238	-9.2
F34	-47.951	-5.655	-9
Z21	-45.916	-7.031	-9.1

Table S4: Glide docking results (continued)

Compound	Prime/MMGBSA energy (kcal/mol)	Glide XP score	AutoDock Vina binding score
TrkA (PDB ID: 4YPS)			
Co-crystallized ligand	-66.737	N/A	N/A
Re-docked co-crystallized ligand	-62.378	-9.321	-9.1
AF3	-62.275	-7.630	-9.5
Z88 (neutral OH/neutral NH)	-59.874/-68.193	-9.550/-9.688	-9.9
Z21	-51.638	-6.229	-9.5
F34	-49.053	-5.117	-10.4

[a]non applicable; [b]R enantiomer of Z88; [c]the core of the co-crystallized compound was used to constrain the docking as the crystal pose could not reproduced by 'free' docking; the alternative pose generated without constraints had a Prime/MMGBSA energy of -50.112 and XP Score of -7.711.

A) Receptor tyrosine kinases

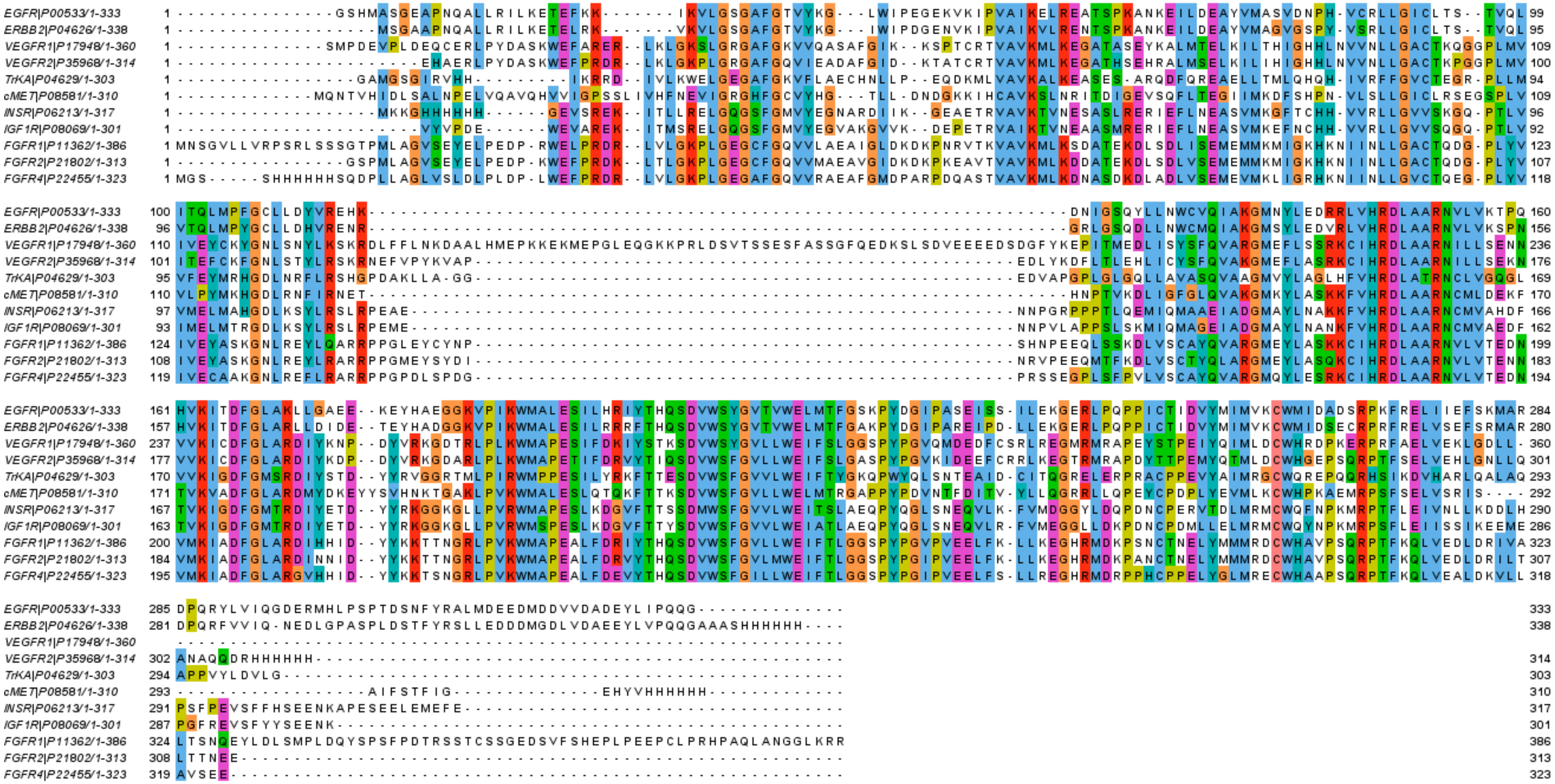

B) Serine/Threonine Kinases

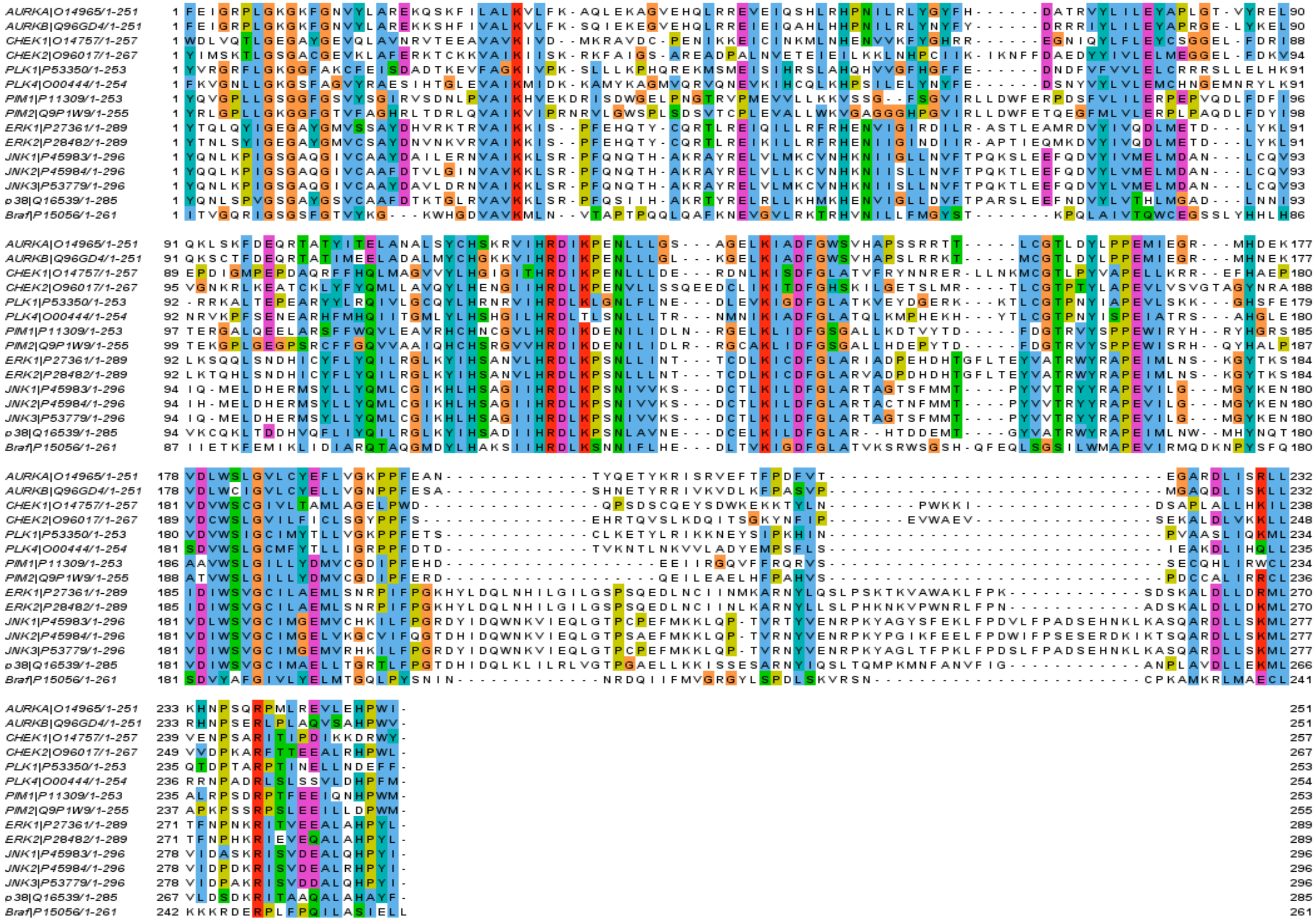

Figure S1: Multiple sequence alignment of some of the kinases used in the study. Conservation of residues are displayed in distinct colors. Numbers on the left represent the starting residue in the alignment. The alignment was generated using Jalview 2.7 [56].

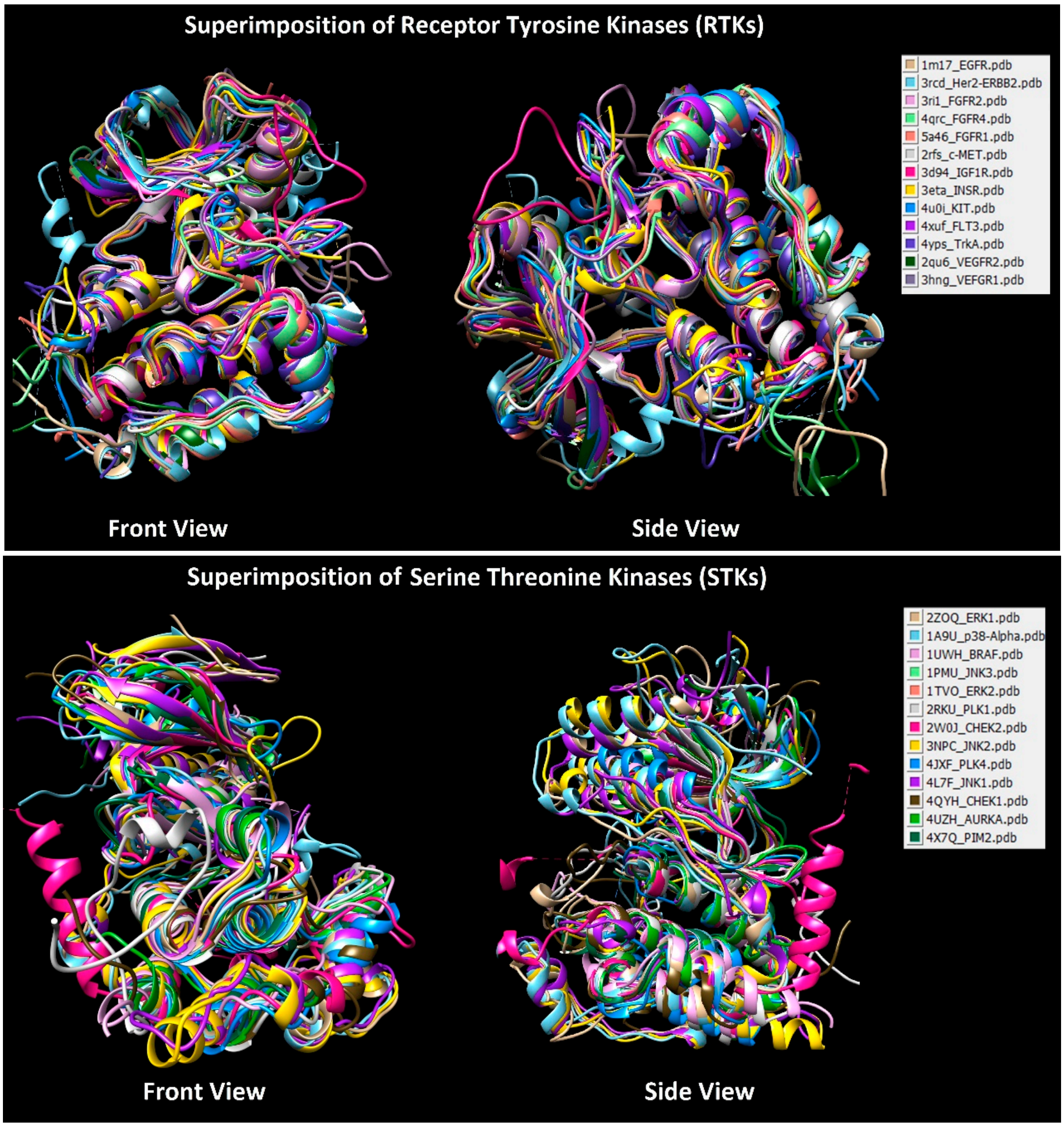

Figure S2: Structural insight in terms of superimposition of RTKs (above panel) and STKs (below panel) are displayed. Each selected RTKs and STKs has shown with different distinct color.

References

1 Stamos, J., Sliwkowski, M.X. & Eigenbrot, C. Structure of the epidermal growth factor receptor kinase domain alone and in complex with a 4-anilinoquinazoline inhibitor. *Journal of Biological Chemistry* **277**, 46265-46272 (2002).

2 Stepanski, E.J. *et al.* Second-and Third-line Treatment of Patients With Non–Small-Cell Lung Cancer With Erlotinib in the Community Setting: Retrospective Study of Patient Healthcare Utilization and Symptom Burden. *Clinical lung cancer* **10**, 426-432 (2009).

3 Ishikawa, T. *et al.* in *ABSTRACTS OF PAPERS OF THE AMERICAN CHEMICAL SOCIETY.* (AMER CHEMICAL SOC 1155 16TH ST, NW, WASHINGTON, DC 20036 USA).

4 Burstein, H., Lieberman, G., Slamon, D., Winer, E. & Klein, P. Isolated central nervous system metastases in patients with HER2-overexpressing advanced breast cancer treated with first-line trastuzumab-based therapy. *Annals of oncology* **16**, 1772-1777 (2005).

5 Yin, M., Guan, X., Liao, Z. & Wei, Q. Insulin-like growth factor-1 receptor-targeted therapy for non-small cell lung cancer: A mini review. *American journal of translational research* **1**, 101 (2009).

6 Wu, J. *et al.* Small-molecule inhibition and activation-loop trans-phosphorylation of the IGF1 receptor. *The EMBO journal* **27**, 1985-1994 (2008).

7 Patnaik, S. *et al.* Discovery of 3, 5-disubstituted-1H-pyrrolo [2, 3-b] pyridines as potent inhibitors of the insulin-like growth factor-1 receptor (IGF-1R) tyrosine kinase. *Bioorganic & medicinal chemistry letters* **19**, 3136-3140 (2009).

8 Heidegger, I., Kern, J., Ofer, P., Klocker, H. & Massoner, P. Oncogenic functions of IGF1R and INSR in prostate cancer include enhanced tumor growth, cell migration and angiogenesis. *Oncotarget* **5**, 2723 (2014).

9 Zorn, J.A., Wang, Q., Fujimura, E., Barros, T. & Kuriyan, J. Crystal structure of the FLT3 kinase domain bound to the inhibitor quizartinib (AC220). *PLoS ONE* **10**, e0121177 (2015).

10 Garner, A.P. *et al.* Ponatinib inhibits polyclonal drug-resistant KIT oncoproteins and shows therapeutic potential in heavily pretreated gastrointestinal stromal tumor (GIST) patients. *Clinical Cancer Research* **20**, 5745-5755 (2014).

11 Gschwind, A., Fischer, O.M. & Ullrich, A. The discovery of receptor tyrosine kinases: Targets for cancer therapy. *Nature Reviews Cancer* **4**, 361 (2004).

12 Klein, T. *et al.* Structural and dynamic insights into the energetics of activation loop rearrangement in FGFR1 kinase. *Nature communications* **6**, 7877 (2015).

13 Zwick, E., Bange, J. & Ullrich, A. Receptor tyrosine kinase signalling as a target for cancer intervention strategies. *Endocrine-related cancer* **8**, 161-173 (2001).

14 Eathiraj, S. *et al.* A novel mode of protein kinase inhibition exploiting hydrophobic motifs of autoinhibited Kinases discovery of atp-independent inhibitors of fibroblast growth factor receptor. *Journal of Biological Chemistry* **286**, 20677-20687 (2011).

15 Tresaugues, L. *et al.* Crystal structure of VEGFR1 in complex with N-(4-Chlorophenyl)-2-((pyridin-4-ylmethyl) amino) benzamide. *The RCSB PDB* (2013).

16 Zwick, E., Bange, J. & Ullrich, A. Receptor tyrosine kinases as targets for anticancer drugs. *Trends in molecular medicine* **8**, 17-23 (2002).

17 Potashman, M.H. *et al.* Design, synthesis, and evaluation of orally active benzimidazoles and benzoxazoles as vascular endothelial growth factor-2 receptor tyrosine kinase inhibitors. *Journal of medicinal chemistry* **50**, 4351-4373 (2007).

18 Ma, P.C., Maulik, G., Christensen, J. & Salgia, R. c-Met: Structure, functions and potential for therapeutic inhibition. *Cancer and Metastasis Reviews* **22**, 309-325 (2003).

19 Shattuck, D.L., Miller, J.K., Carraway, K.L. & Sweeney, C. Met receptor contributes to trastuzumab resistance of Her2-overexpressing breast cancer cells. *Cancer research* **68**, 1471-1477 (2008).

20 Bellon, S.F. *et al.* c-Met inhibitors with novel binding mode show activity against several hereditary papillary renal cell carcinoma-related mutations. *Journal of Biological Chemistry* **283**, 2675-2683 (2008).

21 Nakagawara, A. Trk receptor tyrosine kinases: A bridge between cancer and neural development. *Cancer letters* **169**, 107-114 (2001).

22 Choi, H.-S. *et al.* (R)-2-phenylpyrrolidine substituted imidazopyridazines: A new class of potent and selective pan-TRK Inhibitors. *ACS medicinal chemistry letters* **6**, 562-567 (2015).

23 Battistutta, R. *et al.* Unprecedented selectivity and structural determinants of a new class of protein kinase CK2 inhibitors in clinical trials for the treatment of cancer. *Biochemistry* **50**, 8478-8488 (2011).

24 Sarno, S. *et al.* ATP site-directed inhibitors of protein kinase CK2: An update. *Current topics in medicinal chemistry* **11**, 1340-1351 (2011).

25 Bischoff, J.R. *et al.* A homologue of Drosophila aurora kinase is oncogenic and amplified in human colorectal cancers. *The EMBO journal* **17**, 3052-3065 (1998).

26 Carry, J.-C. *et al.* SAR156497, an exquisitely selective inhibitor of aurora kinases. *Journal of medicinal chemistry* **58**, 362-375 (2014).

27 Elkins, J.M., Santaguida, S., Musacchio, A. & Knapp, S. Crystal structure of human aurora B in complex with INCENP and VX-680. *Journal of medicinal chemistry* **55**, 7841-7848 (2012).

28 Gazzard, L. *et al.* Discovery of the 1, 7-diazacarbazole class of inhibitors of checkpoint kinase 1. *Bioorganic & medicinal chemistry letters* **24**, 5704-5709 (2014).

29 Varmark, H., Kwak, S. & Theurkauf, W.E. A role for Chk2 in DNA damage induced mitotic delays in human colorectal cancer cells. *Cell Cycle* **9**, 312-320 (2010).

30 Lountos, G.T. *et al.* Crystal structure of checkpoint kinase 2 in complex with NSC 109555, a potent and selective inhibitor. *Protein science* **18**, 92-100 (2009).

31 Lei, M. & Erikson, R. Plk1 depletion in nontransformed diploid cells activates the DNA-damage checkpoint. *Oncogene* **27**, 3935 (2008).

32 Takahashi, T. *et al.* Polo-like kinase 1 (PLK1) is overexpressed in primary colorectal cancers. *Cancer science* **94**, 148-152 (2003).

33 Kothe, M. *et al.* Selectivity-determining Residues in Plk1. *Chemical biology & drug design* **70**, 540-546 (2007).

34 Malumbres, M. & Barbacid, M. Cell cycle kinases in cancer. *Current opinion in genetics & development* **17**, 60-65 (2007).

35 Takai, N., Hamanaka, R., Yoshimatsu, J. & Miyakawa, I. Polo-like kinases (Plks) and cancer. *Oncogene* **24**, 287 (2005).

36 Pogacic, V. *et al.* Structural analysis identifies imidazo [1, 2-b] pyridazines as PIM kinase inhibitors with in vitro antileukemic activity. *Cancer research* **67**, 6916-6924 (2007).

37 Guo, S. *et al.* Overexpression of Pim-1 in bladder cancer. *Journal of Experimental & Clinical Cancer Research* **29**, 161 (2010).

38 Narlik-Grassow, M., Blanco-Aparicio, C. & Carnero, A. The PIM family of serine/threonine kinases in cancer. *Medicinal research reviews* **34**, 136-159 (2014).

39 Cibull, T. *et al.* Overexpression of Pim-1 during progression of prostatic adenocarcinoma. *Journal of clinical pathology* **59**, 285-288 (2006).

40 Ishchenko, A. *et al.* Structure-based design of low-nanomolar PIM kinase inhibitors. *Bioorganic & medicinal chemistry letters* **25**, 474-480 (2015).

41 Kinoshita, T. *et al.* Crystal structure of human mono-phosphorylated ERK1 at Tyr204. *Biochemical and biophysical research communications* **377**, 1123-1127 (2008).

42 Duong-Ly, K.C. & Peterson, J.R. The human kinome and kinase inhibition. *Current protocols in pharmacology*, 2.9. 1-2.9. 14 (2013).

43 Capra, M. *et al.* Frequent alterations in the expression of serine/threonine kinases in human cancers. *Cancer research* **66**, 8147-8154 (2006).

44 Blume-Jensen, P. & Hunter, T. Oncogenic kinase signalling. *Nature* **411**, 355 (2001).

45 Ohori, M. *et al.* Identification of a selective ERK inhibitor and structural determination of the inhibitor–ERK2 complex. *Biochemical and biophysical research communications* **336**, 357-363 (2005).

46 Wagner, E.F. & Nebreda, Á.R. Signal integration by JNK and p38 MAPK pathways in cancer development. *Nature Reviews Cancer* **9**, 537 (2009).

47 Fang, J.Y. & Richardson, B.C. The MAPK signalling pathways and colorectal cancer. *The lancet oncology* **6**, 322-327 (2005).

48 Li, B. *et al.* Hit-to-lead optimization and kinase selectivity of imidazo [1, 2-a] quinoxalin-4-amine derived JNK1 inhibitors. *Bioorganic & medicinal chemistry letters* **23**, 5217-5222 (2013).

49 Kuglstatter, A. *et al.* X-ray crystal structure of JNK2 complexed with the p38α inhibitor BIRB796: Insights into the rational design of DFG-out binding MAP kinase inhibitors. *Bioorganic & medicinal chemistry letters* **20**, 5217-5220 (2010).
50 Scapin, G., Patel, S.B., Lisnock, J., Becker, J.W. & LoGrasso, P.V. The structure of JNK3 in complex with small molecule inhibitors: Structural basis for potency and selectivity. *Chemistry & biology* **10**, 705-712 (2003).
51 Wang, Z. *et al.* Structural basis of inhibitor selectivity in MAP kinases. *Structure* **6**, 1117-1128 (1998).
52 Wan, P.T. *et al.* Mechanism of activation of the RAF-ERK signaling pathway by oncogenic mutations of B-RAF. *Cell* **116**, 855-867 (2004).
53 Palanisamy, N. *et al.* Rearrangements of the RAF kinase pathway in prostate cancer, gastric cancer and melanoma. *Nature medicine* **16**, 793 (2010).
54 Lu, H. & Schulze-Gahmen, U. Toward understanding the structural basis of cyclin-dependent kinase 6 specific inhibition. *Journal of medicinal chemistry* **49**, 3826-3831 (2006).
55 Sherr, C.J. Cancer cell cycles. *Science* **274**, 1672-1677 (1996).
56 Brown, N.R. *et al.* CDK1 structures reveal conserved and unique features of the essential cell cycle CDK. *Nature communications* **6**, 6769 (2015).
57 Waterhouse AM, Procter JB, Martin DM, Clamp M, Barton GJ. Jalview Version 2—a multiple sequence alignment editor and analysis workbench. Bioinformatics. 2009 Jan 16;25(9):1189-91.

.
